# Vγ9Vδ2 T cells in tuberculosis: protective immunity and translational perspectives

**DOI:** 10.3389/fimmu.2026.1884056

**Published:** 2026-08-03

**Authors:** Zhiyun Shi, Yixin Ding, Xueyan Jing, Fan Wang, Xuelan Huang, Peng Wang, Hekun Liu, Ningkui Niu

**Affiliations:** 1 Medical Experiment Center, General Hospital of Ningxia Medical University, Yinchuan, China; 2 Key Laboratory of Clinical Pathogenic Microorganisms, General Hospital of Ningxia Medical University, Yinchuan, China; 3 The First Clinical Medical College, Ningxia Medical University, Yinchuan, China; 4 Department of Cell Biology and Genetics, School of Basic Medical Sciences, Fujian Medical University, Fuzhou, China; 5 Department of Orthopedic, General Hospital of Ningxia Medical University, Yinchuan, China; 6 Research Center for Prevention and Control of Bone and Joint Tuberculosis, General Hospital of Ningxia Medical University, Yinchuan, China

**Keywords:** butyrophilins, immune memory, immunotherapy, innate-like T cells, mycobacterium tuberculosis, phosphoantigens, tuberculosis vaccine, Vγ9Vδ2 T cells

## Abstract

**Background:**

Vγ9Vδ2 T cells constitute the predominant human circulating γδ T cell subset and serve as key mediators of anti-mycobacterial immunity through major histocompatibility complex (MHC)-unrestricted phosphoantigen recognition and rapid effector activation.

**Summary:**

This review evaluates Vγ9Vδ2 T cell biology in the context of tuberculosis (TB). We delineate the three-stage developmental pathway of Vγ9Vδ2 T cells in the postnatal thymus and the butyrophilin 3A1/2A1 (BTN3A1/BTN2A1)-mediated “inside-out” antigen sensing mechanism, noting the limitations of extrapolating *in vitro* findings to *in vivo* TB infection. The multifaceted activation networks—involving TCR-dependent signaling, cytokine amplification, and accessory co-receptor pathways—are examined in relation to distinct phases of *Mtb* infection. Vγ9Vδ2 T cells exert anti-TB protection through direct cytotoxicity (perforin/granzyme B, granulysin, FasL/TRAIL), cytokine-mediated immunoregulation, and orchestration of αβ T cell and dendritic cell responses; however, the functional significance of granulysin-dependent killing and the paradoxical Vδ2→Vδ1 subset shift during chronic infection warrant further investigation. *Mtb* immune evasion through metabolic antigen camouflage and chronic infection-induced exhaustion is also analyzed. Emerging evidence for Vγ9Vδ2 T cell immunological memory—including BCG-induced trained immunity and phosphoantigen-driven memory-like expansion in non-human primates —provides a rationale for novel vaccine strategies, although the translatability of these findings to human TB remains to be established. Translational approaches, including synthetic phosphoantigen prodrugs, BTN-targeted monoclonal antibodies, and adoptive cell therapy, are assessed for their clinical potential against drug-resistant TB, with discussion of current limitations.

**Conclusion:**

Vγ9Vδ2 T cells offer distinct advantages for TB immunotherapy and vaccine design, but significant translational barriers remain. Future studies must address subset heterogeneity across infection stages, the epigenetic and metabolic reprogramming governing functional fate decisions, standardized correlates of protection, and BTN-targeted strategies validated in TB-specific preclinical models. The integration of single-cell multi-omics and CRISPR-based functional screening with γδ T cell biology offers a promising path toward clinical translation.

## Introduction

Tuberculosis (TB) is a highly contagious airborne infectious disease caused by *Mycobacterium tuberculosis* (*Mtb*), posing a significant threat to human health. According to the World Health Organization (WHO) Global Tuberculosis Report 2025, the annual mortality from TB has nearly doubled that of HIV/AIDS, underscoring its persistent global health burden (1, 2). Despite the availability of vaccines and antibiotics, the emergence of drug-resistant strains and the syndemic with HIV have substantially complicated TB control efforts (1, 2). The host immune response to *Mtb* is essential for infection containment, and γδ T cells play a pivotal role in this process. γδ T cells represent a non-conventional subset of lymphocytes that exhibit characteristics of both innate and adaptive immunity, serving as a functional bridge in anti-infectious immune responses (3). Vγ9Vδ2 T cells constitute the predominant γδ T cell subset in peripheral blood and can be rapidly activated upon recognition of phosphorylated antigens (e.g., HMB-PP) produced by *Mtb*, thereby exerting anti-infective functions (4). Recent studies have demonstrated that Vγ9Vδ2 T cells not only participate in early immune responses during *Mtb* infection but also possess immunological memory and immunomodulatory properties (5, 6), offering novel therapeutic targets for immunotherapy and vaccine development against TB. This review summarizes the biological characteristics, activation mechanisms, immunoprotective roles against *Mtb* infection, immunological memory formation, and potential therapeutic strategies of Vγ9Vδ2 T cells, with particular attention to translational barriers and unresolved questions that constrain progress toward clinical implementation.

Cells of the innate immune system, such as dendritic cells (DCs) and macrophages, express a broad repertoire of pattern recognition receptors (PRRs) that recognize pathogen-associated molecular patterns (PAMPs) during infection and damage-associated molecular patterns (DAMPs) released during tissue injury, infection, and malignancy. Cells constituting the adaptive immune system undergo genetic recombination and express specific antigen receptors, such as the αβ T cell receptor (TCR), or secrete antibodies, such as B cells (7). T cell lineages are defined by specialized functions and differential expression of surface antigens, cytokines, and transcription factors. The most extensively studied are conventional T cells, which express the αβ TCR and recognize peptide antigens presented by major histocompatibility complex (MHC) molecules; unconventional T cells, including natural killer T (NKT) cells, mucosal-associated invariant T (MAIT) cells, and γδ T cells, recognize non-peptide antigens such as lipids, vitamin B metabolites, and phosphorylated antigens, respectively, accounting for approximately 10% of circulating T cells in humans. These cell lineages typically express TCRs with limited diversity and have been characterized as cells that bridge innate and adaptive immunity (8).

**Figure 1:**
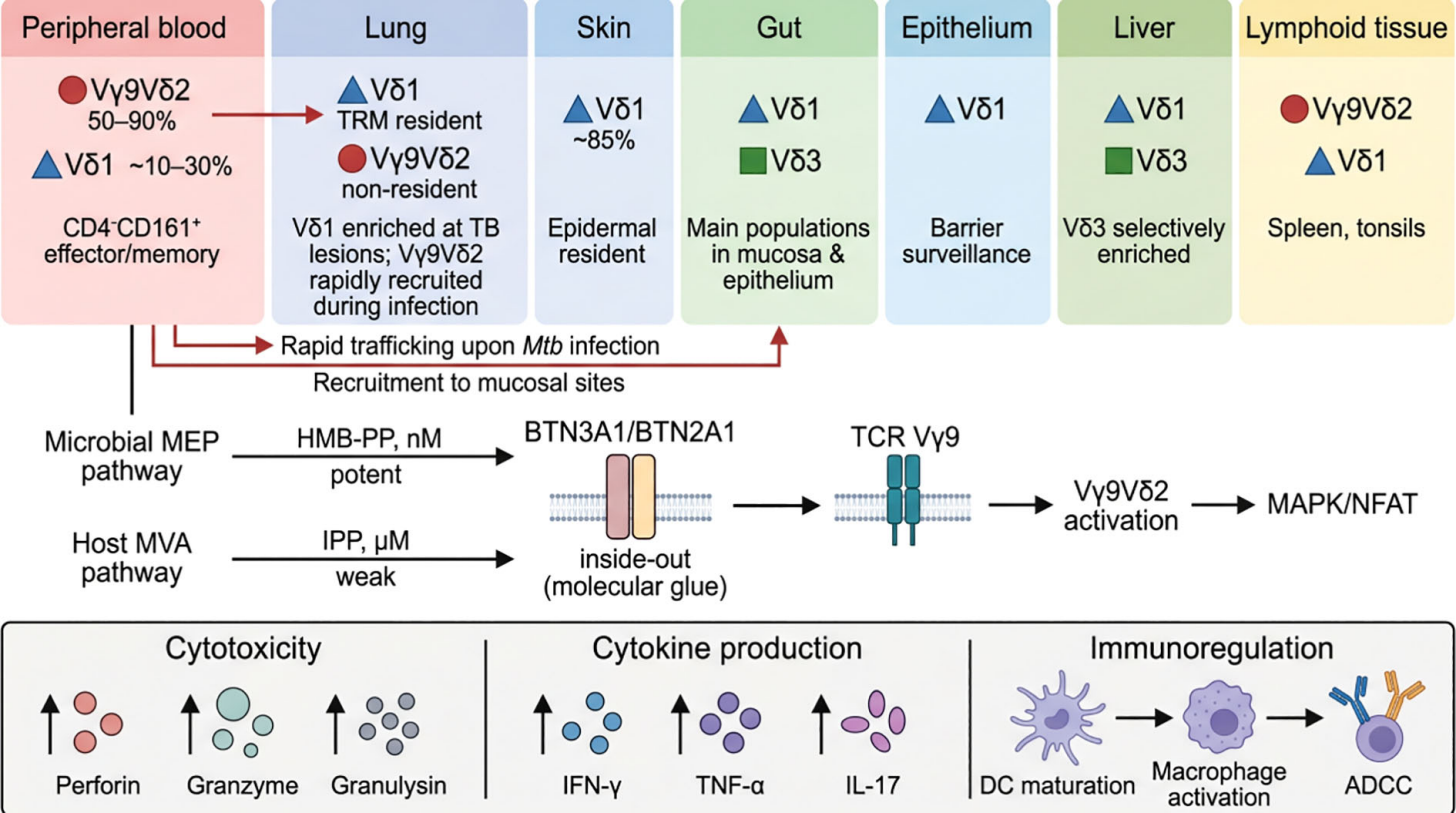
Schematic diagram of tissue distribution and phosphoantigen sensing of human γδ T cell subsets. Human γδ T cell subsets exhibit distinct tissue distribution patterns. Vγ9Vδ2 T cells predominate in peripheral blood (50–90% of circulating γδ T cells), while Vδ1^+^ γδ T cells are enriched in skin, lung, and epithelial tissues as tissue-resident populations. Vδ3^+^ γδ T cells are enriched in the liver and intestinal epithelium. In peripheral blood, Vγ9Vδ2 T cells sense phosphoantigens (pAgs) derived from the microbial methylerythritol phosphate (MEP; non-mevalonate) pathway—primarily (E)-4-hydroxy-3-methyl-but-2-enyl pyrophosphate (HMB-PP)—or from the host mevalonate (MVA) pathway—isopentenyl pyrophosphate (IPP)—via butyrophilin 3A1/2A1 (BTN3A1/BTN2A1)-mediated inside-out activation, leading to downstream signaling through the MAPK/NFAT axis and effector responses including cytotoxicity (perforin, granzyme, granulysin), cytokine production (interferon-γ [IFN-γ], tumor necrosis factor-α [TNF-α]), and immunoregulatory crosstalk with dendritic cells (DCs) and macrophages. Notably, Vδ1^+^ γδ T cells are enriched at tuberculosis (TB) granuloma sites in the lung as tissue-resident cells, while Vγ9Vδ2 T cells are rapidly recruited from circulation upon *Mycobacterium tuberculosis* (*Mtb*) infection. Vδ1 and Vδ3 constitute the main γδ T cell populations in the intestinal mucosa and epithelium. Assisted generation via jova.ai; edited, revised, and finalized by the authors.

γδ T cells are tissue-resident innate-like T cells present in organized lymphoid tissues, with higher frequencies observed in tissues such as the skin, intestine, and liver (9). Based on the δ chain, human γδ T cells are classified into three major subsets: Vδ1, Vδ2, and Vδ3. Among these, Vγ9Vδ2 T cells represent the predominant circulating γδ T cell subset in humans, are primarily found in peripheral blood, and are typically CD4^−^CD8^−^, expressing CD161 as well as memory and effector cell markers (10). The Vγ9Vδ2 T cell receptor (TCR) heterodimer is composed of a TCR γ chain utilizing the Vγ9 segment and a TCR δ chain employing the variable (V) segment Vδ2 (11). These cells respond to phosphoantigens (phosphorylated metabolites of eukaryotic and microbial origin) and are potent producers of IFN-γ, perforin, and granzymes (12).The phosphoantigens (pAgs) recognized by Vγ9Vδ2 T cells may be intracellular metabolites of the eukaryotic mevalonate (MVA) pathway, such as isopentenyl pyrophosphate (IPP), which accumulates intracellularly during tumorigenesis; or metabolites derived from the methylerythritol phosphate pathway (MEP) of microbial pathogens, including the non-mevalonate intermediate (E)-4-hydroxy-3-methyl-but-2-enyl pyrophosphate (HMB-PP), with HMB-PP being the most potent stimulus for Vγ9Vδ2 T cells (13) (**Figure 1**).

Notably, phosphoantigens are not directly presented to Vγ9Vδ2 T cells by infected or malignant cells (14). Instead, phosphoantigens bind to the intracellular B30.2 domain of butyrophilin 3A1 (BTN3A1), inducing its association with the B30.2 domain of butyrophilin 2A1 (BTN2A1) (15, 16).This interaction triggers a conformational change in BTN transmembrane proteins, enabling BTN3A1 to dissociate from the extracellular domain of BTN2A1 and facilitating the binding of BTN2A1 to the TCR Vγ9 chain on Vγ9Vδ2 T cells, thereby initiating TCR-mediated γδ T cell activation.

Importantly, rodents lack homologous γδ TCRs that can be activated by phosphoantigens; consequently, conventional murine models are unsuitable for investigating the mechanistic roles of phosphoantigen-reactive γδ T cells in cancer and infectious diseases. However, the development of humanized mouse models has provided appropriate experimental systems for studying human γδ T cells (17). Recently, Vγ9Vδ2 T cells responsive to phosphoantigens in a BTN3-dependent manner were identified in alpacas, exhibiting canonical TRGV9 and TRDV2-like rearrangements, rendering them the first non-primate species characterized with this distinctive feature (18).

Vγ9Vδ2 T cells participate in anti-infectious defense, tumor immune surveillance, and immunoregulatory processes, playing pivotal roles in effective immunity against a broad spectrum of cellular pathogens, including *Mycobacterium tuberculosis*, *Plasmodium* spp., *Listeria monocytogenes*, *Staphylococcus aureus*, and various viral pathogens. For instance, during the acute phase of bacterial and parasitic infections, peripheral blood Vγ9Vδ2 T cells can expand to constitute up to 20–50% of circulating T cells, exerting essential protective functions (19). However, during active TB, circulating Vδ2 T cells may instead become depleted (20). Following bacillus Calmette-Guérin (BCG) vaccination, γδ T cells undergo significant expansion, with clonotypes expressing shared (public) Vγ9-JP and Vδ2 TCR sequences—that is, identical TCR sequences found across genetically unrelated individuals—potentially associated with protective immune responses induced by BCG or *Mtb* infection across ethnically and geographically diverse populations (21). Novel candidate vaccines, such as MTBVAC, can induce the expansion of γδ T cells with a distinct cytotoxic phenotype (CD16^+^GZMB^+^) during early immune responses, a profile resembling effector cells identified in *Mtb* infection controllers (22). A study evaluating a candidate *Plasmodium* vaccine in a human cohort established a correlation between circulating Vγ9Vδ2 T cell frequency and protective immunity (23). Vγ9Vδ2 T cells may also contribute to antiviral immunity (23–26), including against HIV, influenza, Ebola, dengue, and SARS-CoV-2. Di et al. recently reviewed the current knowledge regarding Vγ9Vδ2 T cell-mediated anti-Mtb immunity and the latest advances in vaccine development (27). However, this review primarily focused on the translational potential of Vγ9Vδ2 T cell-based vaccine strategies, without in-depth analysis of BTN-mediated antigen recognition, multi-layered activation signaling networks, or *Mtb* immune evasion mechanisms. This review summarizes the biological characteristics, activation mechanisms, immunoprotective roles against *Mtb* infection, immunological memory formation, and potential therapeutic strategies of Vγ9Vδ2 T cells, with particular attention to translational barriers and unresolved questions that constrain progress toward clinical implementation.

## The biological basis of Vγ9Vδ2 T cell-mediated anti-tuberculosis protection

Vγ9Vδ2 T cells markedly differ from conventional MHC-restricted αβ T cells with respect to thymic development, antigen recognition, and ligand selection. Whereas αβ T cells recognize peptide antigens presented by major histocompatibility complex (MHC) molecules, Vγ9Vδ2 T cells do not require MHC-mediated antigen presentation; instead, they directly recognize non-peptide antigens, participate in both innate and adaptive immune responses (28), and are predominantly found in peripheral blood. Although broader γδ T cell populations are abundant in mucosal tissues such as the skin, intestine, and lung, Vγ9Vδ2 T cells can also migrate to mucosal sites during infection (27), enabling rapid pathogen responsiveness. During thymic development, the strength of TCR–ligand interactions in γδ T cells does not result in classical positive or negative selection; rather, it directs differentiation into IFN-γ–or IL-17–producing cells (29). The majority of phosphoantigen-reactive clones possess a conserved hydrophobic residue at position 97 within the complementarity-determining region 3 (CDR3) of Vδ2, encoded by N-nucleotide additions. Several butyrophilin (BTN) and butyrophilin-like (BTNL) molecules, including BTN2A1, BTN3A1, skint-1, BTNL1, and BTNL6, engage with the TCR (30–32), governing γδ T cell development, activation, and homeostasis.

### Developmental ontogeny and tissue distribution

**Figure 2:**
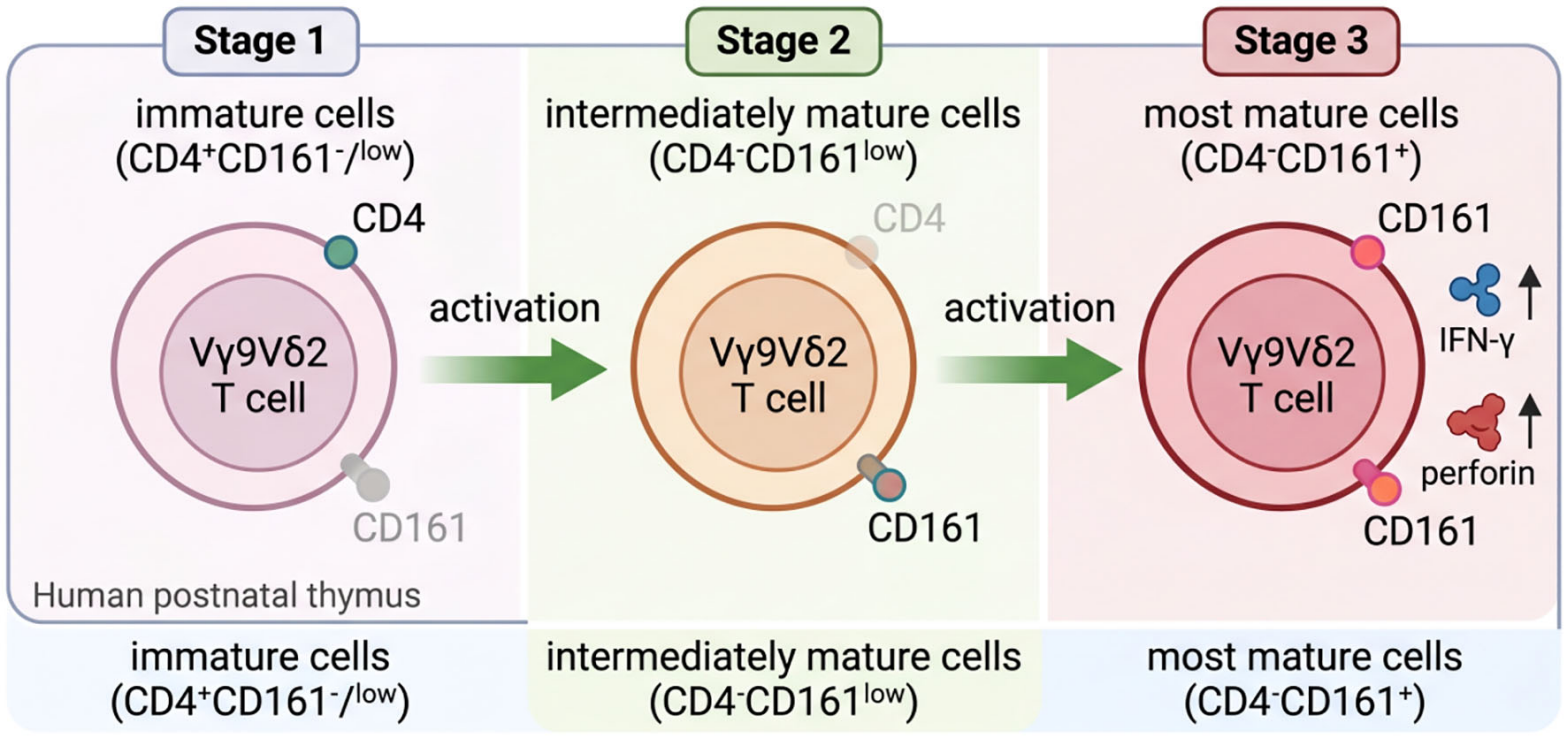
Three-stage developmental pathway of Vγ9Vδ2 T cells in the human postnatal thymus. Stage 1: immature cells (CD4^+^CD161^-^/^low^); Stage 2: intermediately mature cells (CD4^-^CD161^low^); Stage 3: most mature cells (CD4^-^CD161^+^). Mature thymic Vγ9Vδ2 T cells express high levels of interferon-γ (IFN-γ) and perforin. CD, cluster of differentiation. Assisted generation via jova.ai; edited, revised, and finalized by the authors.

The development of γδ T cells encompasses multiple subsets and distinct genetic programs, influenced by the intricate interplay of TCR gene rearrangement, signaling networks, and interactions with the thymic microenvironment. Thymic stromal cells play a pivotal role in γδ T cell development and maturation through various signaling pathways and cellular interactions. TCR repertoire sequencing analyses of blood and thymic Vγ9Vδ2 T cells from fetal to adult stages have revealed that adult Vγ9Vδ2 T cells express TCRs shared with postnatal thymocytes, with identical TCR J segments between postnatal thymic and adult blood Vγ9Vδ2 T cells, which differ from those of fetal blood Vγ9Vδ2 T cells (33). A recent study employing single-cell RNA sequencing (scRNA-seq) combined with high-dimensional flow cytometry provided an in-depth analysis of the developmental trajectory of human thymic Vγ9Vδ2 T cells, defined by the expression of transcription factors, chemokines, and surface markers across three stages: immature cells (CD4^+^CD161^-/low^), intermediately mature cells (CD4^-^CD161^low^), and most mature cells (CD4^−^CD161^+^) (**Figure 2**). This study established that the development of functional Vγ9Vδ2 T cells occurs predominantly in the postnatal thymus rather than in the periphery, and demonstrated that mature thymic Vγ9Vδ2 T cells express high levels of IFN-γ and perforin (12), thereby advancing strategies for harnessing Vγ9Vδ2 T cells in the treatment of human diseases such as cancer and infection.

However, a key limitation of the current understanding of Vγ9Vδ2 T cell ontogeny is the near-exclusive reliance on thymic samples from limited donor cohorts, which may not capture the full spectrum of inter-individual variation. Notably, the functional significance of the observed differences between fetal and adult TCR repertoires remains incompletely understood. Fetal-derived Vγ9Vδ2 T cells harbor public TCR sequences with limited junctional diversity (34), raising the unresolved question of whether these cells provide qualitatively distinct protection compared to their adult counterparts during neonatal *Mtb* exposure. TCR repertoire analyses have revealed that fetal-derived Vγ9Vδ2 T cells undergo pathogen-specific selective expansion in young children with bacterial sepsis, particularly in response to HMBPP-negative *S. aureus*, suggesting that the developmental origin of Vγ9Vδ2 T cells may imprint lasting functional programs (35). Yet, whether thymic involution with age compromises the replenishment of functionally competent Vγ9Vδ2 T cells—and whether this contributes to increased TB susceptibility in elderly populations—remains an important area requiring systematic investigation.

The efficient selection and expansion of γδ T cells underscore their pivotal role in immune responses against infected or malignant cells, as well as their involvement in immunoregulation (36). Postnatal extrathymic selection results in an increased proportion of phosphoantigen-reactive T cells harboring Vγ9-JP rearrangements, comprising approximately 1 in 40 CD45RO+ circulating memory T cells from healthy adults in high-burden TB-endemic settings, although this frequency may vary substantially across populations with different TB exposure histories and HLA backgrounds (37, 38). This subset can undergo rapid clonal expansion in response to infection or malignancy, constituting one of the largest antigen-specific memory T cell pools in humans (38).

From the perspective of tissue distribution, Vγ9Vδ2 T cells are predominantly present in peripheral blood but also exhibit substantial distribution in mucosal tissues such as the lung, a characteristic that enables their rapid response at the frontline of *Mtb* infection. High-throughput TCR sequencing technology has been employed to analyze the distribution frequency and characteristics of γδ TCR repertoires in lung tissue and blood from tuberculosis patients, revealing a marked skewing toward the Vδ1 subset within the δ TCR repertoire in the lung (20). Considerable heterogeneity in γδ TCR repertoires was observed across different lung samples from the same individual, with only 7% of δ TCR clonotypes detectable in the blood. TB-specific γδ T cells demonstrate a pronounced propensity to migrate to and reside within lung tissue, conferring protective activity upon γδ T cells (39). Similarly, in a nonhuman primate model, phosphoantigen-specific Vγ9Vδ2 T cells have been shown to rapidly traffic to the airways and pulmonary compartment following intravenous adoptive transfer, displaying central/effector memory and effector functions capable of producing anti-*Mtb* cytokines (40).

A major unresolved question concerns the limited representation of Vγ9Vδ2 T cells within the lung γδ TCR repertoire relative to Vδ1 T cells. While Vγ9Vδ2 T cells are rapidly recruited from circulation upon *Mtb* infection, the lung microenvironment appears to favor Vδ1 T cell residency—a phenomenon that may reflect distinct tissue-imprinting programs or competitive exclusion within the granulomatous niche. Recent paired single-cell and spatial transcriptional profiling of human TB granulomas has begun to map the cellular architecture of these lesions at unprecedented resolution, revealing compartmentalized immune cell distributions (41); however, γδ T cell subsets were not specifically resolved in these analyses. Spatial transcriptomics combined with multiplexed imaging should be applied to characterize Vγ9Vδ2 versus Vδ1 T cell positioning within and around TB granulomas—an analysis that has not yet been reported.

### Mechanisms of antigen recognition

Vγ9Vδ2 T cells specifically recognize immunogenic phosphoantigens (pAgs) through a novel “inside-out” mechanism involving BTN3A1 and BTN2A1, thereby sensitively detecting metabolites of intracellular pathogens including *Mtb*. Natural phosphoantigens from mycobacteria include isopentenyl pyrophosphate, prenyl pyrophosphate derivatives, and phosphorylated nucleotides (42). Phosphorylated non-peptide antigens (TUBag1 to -4) isolated from lysates of the *Mtb* H37Rv strain were identified as the predominant and potent stimuli for human Vγ9Vδ2 T cell responses to mycobacteria (42, 43). Shortly following the isolation of TUBag1 to -4, the structurally characterized Vγ9Vδ2 T cell antigen was identified as IPP, a metabolite synthesized via two mutually exclusive pathways: the mevalonate pathway in humans and other eukaryotes, and the methylerythritol phosphate (MEP) pathway in *Mtb* and many other bacterial pathogens (44). Notably, HMB-PP, the most potent natural phosphoantigen, is produced exclusively via the MEP pathway, making it a highly sensitive surrogate marker for the immune system to detect foreign infection. However, the concentration of IPP in bacterial lysates is insufficient to stimulate Vγ9Vδ2 T cell expansion (45). Currently, the most potent natural phosphoantigen described is HMB-PP, a phosphorylated intermediate produced by bacteria and certain eukaryotes, which exhibits 10,000- to 30,000-fold greater stimulatory activity than IPP (46). Furthermore, studies have demonstrated that mycobacteria contain a component functioning as a potent mitogen for human γδ T cells, characterized by protease resistance and a low molecular weight (1–3 kDa) carbohydrate-containing nature; unlike classical superantigens that bind MHC class II molecules and polyclonally activate αβ T cells, this component activates γδ T cells through MHC-independent mechanisms, accounting for the active participation of γδ T cells in mycobacterial infection (43). Synthetic monoalkyl phosphates, such as monoethyl phosphate, share chemical similarities with natural mycobacterial ligands and selectively activate Vγ9Vδ2 T cells while stimulating there *in vitro* proliferation, playing a significant role in human immunity against pathogens (47). Notably, the abbreviation MEP is avoided here to prevent confusion with the methylerythritol phosphate (MEP/non-mevalonate) pathway discussed above. During pathogen infection, phosphoantigens initiate massive expansion of the Vγ9Vδ2 T cell population, ultimately resulting in up to 50% of human blood T cells becoming Vγ9Vδ2 T cells (19, 48).

BTN proteins constitute a family of transmembrane proteins belonging to the immunoglobulin superfamily, exhibiting sequence similarity to the costimulatory receptor B7 family (e.g., CD80, PD-L1). BTN proteins engage with the Vγ9Vδ2 TCR to modulate T cell activation and also serve as intracellular sensors for phosphoantigens (e.g., IPP and HMB-PP), thereby possessing broad immunomodulatory functions. Human BTN genes are located within a gene cluster at the telomeric end of the major histocompatibility complex (MHC) on chromosome 6 (49). The proteins encoded by BTN2A1, BTN3A1 (CD277), BTN3A2, and BTN3A3 contribute to γδ T cell activation (50, 51). BTN3A1 is recognized as the primary ligand for human Vγ9Vδ2 T cell recognition; its gene expression exhibits a certain degree of polymorphism, and BTN3A1/CD277 plays a pivotal role in the antigen-mediated activation of human γδ T cells (52). Rhodes et al. constructed single-knockout cell lines for BTN3A1, BTN3A2, and BTN3A3, respectively, and demonstrated that BTN3A2-knockdown target cells exhibited defects in mediating pAg-induced T cell activation comparable in severity to those observed with BTN3A1 knockdown, revealing that BTN3A2 and BTN3A3 family members also make substantial contributions to T cell activation (53). Studies have shown that BTN2A1 can directly interact with the Vγ9Vδ2 TCR, and a synergistic relationship exists between BTN2A1 and BTN3A1 in coordinately regulating Vγ9Vδ2 T cell function (54). Uldrich et al. (55), through structural and functional analyses of BTN2A1 and BTN3A1, identified that the interaction between BTN2A1 and BTN3A1 serves an important regulatory role in γδ T cell immunological recognition of pAgs, and proposed a novel paradigm for phosphoantigen recognition.

Despite these mechanistic advances, several key questions regarding BTN-mediated antigen recognition remain unresolved. First, while BTN3A1 gene polymorphism has been noted (52), the functional significance of BTN3A1 polymorphic variants in the context of TB susceptibility has not been systematically investigated. Given that BTN3A1 is the essential intracellular sensor for phosphoantigens, genetic variants that alter B30.2 domain conformation or pAg-binding affinity could theoretically modulate individual susceptibility to TB—a hypothesis supported by the known association of BTN3A1 polymorphisms with systemic lupus erythematosus (SLE) risk (56) but untested in TB cohorts. Second, the mechanism by which non-phosphorylated antigens (such as mycobacterial protein antigens DXS2, OxyS, and Rv2272 EP) activate Vγ9Vδ2 T cells independently of BTN molecules remains poorly characterized at the molecular level. These antigens reportedly engage the γδ TCR directly (57), yet the structural basis for this BTN-independent recognition pathway has not been resolved crystallographically. Third, recent findings demonstrating that the synthetic MEP pathway multi-gene construct failed to enhance Vγ9Vδ2 T cell expansion in recombinant BCG due to metabolic feedback inhibition (58) raise important questions about the natural regulation of HMB-PP production by *Mtb*. Specifically, the mechanisms by which *Mtb* precisely calibrates HMB-PP synthesis under different microenvironmental conditions—including hypoxia, nutrient deprivation, and intracellular stress within macrophages—remain largely unexplored (58), representing a significant gap in our understanding of host-pathogen metabolic interactions.

**Figure 3:**
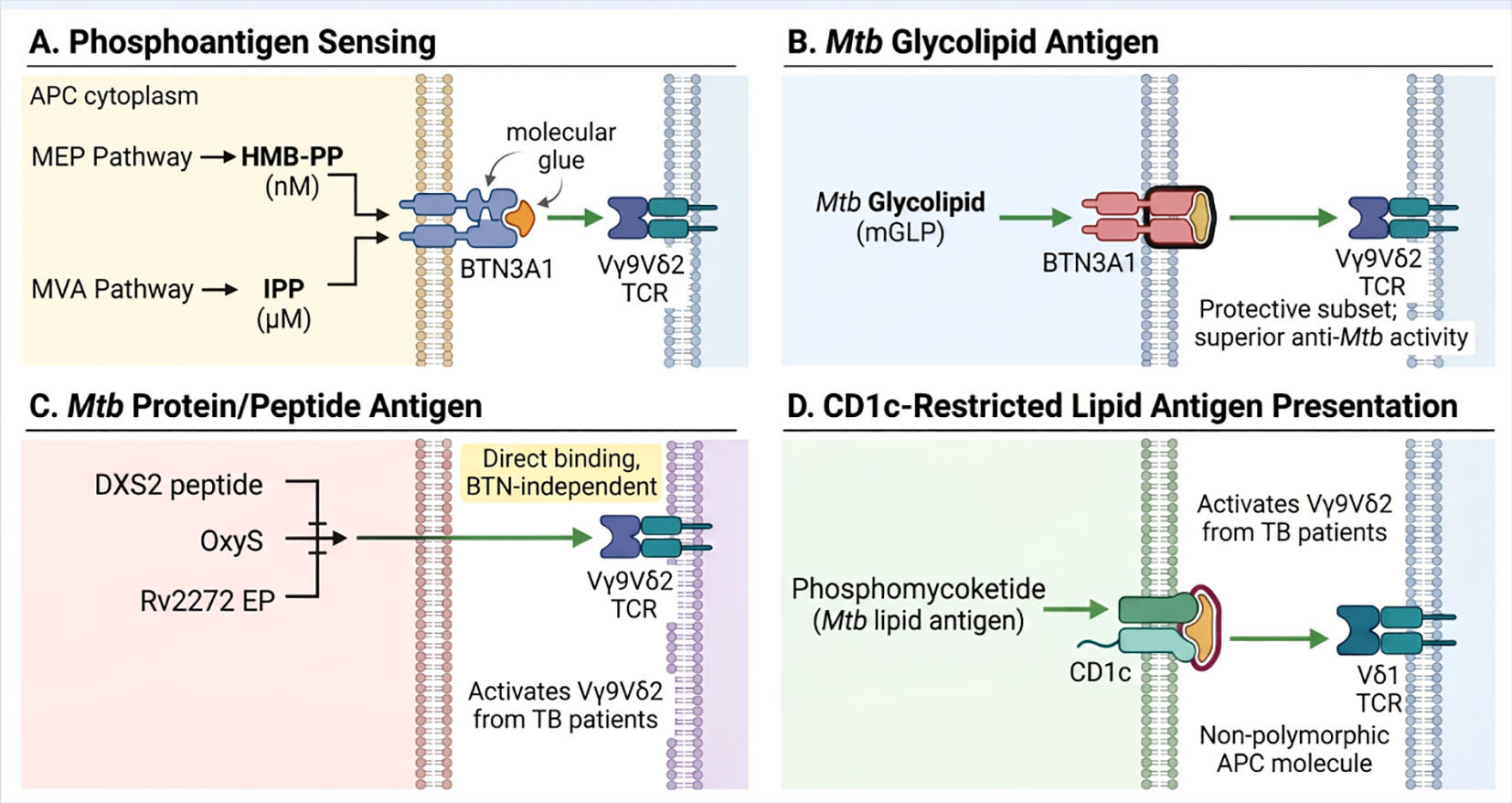
Antigen recognition pathways of human γδ T cells in tuberculosis. Four distinct pathways mediate γδ T cell activation by *Mycobacterium tuberculosis* (*Mtb*) antigens. **(A)** Vγ9Vδ2 T cells sense phosphoantigens (pAgs)—(E)-4-hydroxy-3-methyl-but-2-enyl pyrophosphate (HMB-PP) from the microbial methylerythritol phosphate (MEP) pathway and isopentenyl pyrophosphate (IPP) from the host mevalonate (MVA) pathway—via butyrophilin 3A1/2A1 (BTN3A1/BTN2A1)-mediated inside-out activation. **(B)** A mycobacterial glycolipid, 6-O-methylglucose lipopolysaccharide (mGLP), activates a protective subset of Vγ9Vδ2 T cells through BTN3A1-dependent but phosphoantigen-independent mechanisms; mGLP-expanded Vγ9Vδ2 T cells exhibit superior anti-mycobacterial activity compared to HMB-PP-expanded cells (60). **(C)** Mycobacterial protein/peptide antigens—including 1-deoxy-D-xylulose 5-phosphate synthase 2 (DXS2) peptide, oxidative stress response regulatory protein (OxyS), and Rv2272 extracellular peptide (EP)—directly engage the γδ T cell receptor (TCR) independently of BTN molecules, activating Vγ9Vδ2 T cells from TB patients to produce interferon-γ (IFN-γ) and monocyte chemoattractant protein-1 (MCP-1) (57). **(D)** Mycobacterial phosphomycoketide antigens are presented by CD1c to Vδ1^+^ γδ T cells, representing a CD1-restricted lipid antigen recognition pathway distinct from Vγ9Vδ2-mediated phosphoantigen sensing (61). TB, tuberculosis. Assisted generation via jova.ai; edited, revised, and finalized by the authors.

In addition to phosphoantigens, Vγ9Vδ2 T cells can recognize certain non-phosphorylated antigens. In the context of infected cells, superantigens such as staphylococcal enterotoxin A (59) and lipid components of *Mtb* have been identified as specific activators of Vγ9Vδ2 T cells. Several studies have characterized novel mycobacterial protein antigens recognized by human γδ TCR-bearing cells, including the oxidative stress response regulatory protein (OxyS) (57), 1-deoxy-D-xylulose 5-phosphate synthase 2 (DXS2), and the Rv2272 extracellular peptide (EP), which can activate γδ T cells from BCG-vaccinated human subjects and mediate cytotoxic effects against mycobacteria. Xia et al. employed mass spectrometry analysis and immunological imaging techniques to investigate tuberculosis-associated antigens capable of inducing protective Vγ9Vδ2 T cell expansion; specifically, 6-O-methylglucose lipopolysaccharide (mGLP), a polar glycolipid component of the mycobacterial cell wall, was found to stimulate a protective subset of Vγ9Vδ2 T cells in a BTN3A1-dependent but phosphoantigen-independent manner, and effectively inhibit intracellular *Mtb* growth (60), potentially offering novel immunotherapeutic strategies for both drug-susceptible and drug-resistant tuberculosis. The *Mtb* cell wall is enriched in lipids, and these lipid antigens can be recognized by γδ T cells. For instance, *Mycobacterium tuberculosis* phosphomycoketide represents a lipid molecule presented by CD1c to Vδ1 TCR-expressing γδ T cells (61), constituting yet another independent antigen recognition pathway. Consequently, mGLP and mycobacterial ketides appear to serve as specific antigens suitable for incorporation into tuberculosis vaccines (**Figure 3**).

### Networks governing activation and regulation

The activation of Vγ9Vδ2 T cells represents a complex process involving diverse cell types, molecules, and signaling pathways (62). These cells exert multifaceted functions in anti-tuberculosis immune responses, including direct killing of infected cells, immunomodulation, and immunological memory formation. Notably, Vγ9Vδ2 T cells can respond to both TCR-dependent and TCR-independent stimuli; for instance, CD molecules, Toll-like receptors (TLRs), costimulatory molecules, natural killer receptors (NKG2D), and cytokines can enhance their reactivity (63).

### TCR-dependent signaling

Phosphoantigens trigger a cascade of signaling events in Vγ9Vδ2 T cells. At the TCR-proximal level, IPP induces rapid and sustained activation of PKCθ, as well as phosphorylation of extracellular signal-regulated kinases 1/2 (ERK1/2), p38 mitogen-activated protein kinase (p38 MAPK), and c-Jun N-terminal kinase (JNK), leading to activation of nuclear factor-kappa B (NF-κB) and activator protein-1 (AP-1), and subsequent release of IFN-γ and TNF-α (64). Downstream of TCR engagement, HMB-PP induces rapid and sustained MEK/Erk and PI-3K/Akt signal transduction, governing Vγ9Vδ2 T cell activation, proliferation, and cytotoxicity (65). Li et al. further confirmed that MEK/Erk and PI-3K/Akt activation is requisite for inducing effector responses, and that pAg-induced calcium mobilization and nuclear factor of activated T cell (NFAT) translocation are necessary for activating the effector functions of Vγ9Vδ2 T cells (66). Beyond TCR-proximal signals, cytokine pathways amplify Vγ9Vδ2 T cell responses: IL-12 amplifies HMB-PP-activated Vγ9Vδ2 T cells through PI3K/AKT and STAT4 activation, as well as endogenous TNF-α signaling, and inhibits intracellular mycobacterial growth in an IFN-γ– or TNF-α–dependent manner (67); the synergistic effect of IL-15 plus IL-12 results in dual activation of STAT1 and STAT4, thereby enhancing IFN-γ production by γδ T cells (68). Ribot et al. (69) analyzed human pediatric thymus and found that ex vivo γδ thymocytes differentiate into cytotoxic type 1 effector T cells secreting perforin and IFN-γ following IL-2/IL-15 signaling. Additionally, γδ T cells express Notch1 and Notch2 at both mRNA and protein levels; knockdown of Notch1 and Notch2 suppresses their antitumor cytotoxic potential, delineating Notch as an accessory signal that facilitates antigen-specific effector functions of γδ T cells (70) (**Table 1**).

**Table 1:** Signaling networks governing Vγ9Vδ2 T cell activation and regulation.

Category	Signal/ligand	Receptor/sensor	Key downstream pathway	Functional outcome	Reference
TCR-dependent signaling	HMB-PP/IPP (intracellular pAgs)	BTN3A1 (B30.2 intracellular domain) → BTN2A1 → Vγ9 TCR	Inside-out conformational change; BTN2A1–Vγ9TCR binding initiates TCR signaling	TCR-mediated Vγ9Vδ2 T cell activation; phosphoantigen sensing	(15, 16, 53, 54)
	IPP	Vγ9Vδ2 TCR (proximal)	PKCθ activation; ERK1/2, p38 MAPK, JNK phosphorylation → NF-κB + AP-1	IFN-γ and TNF-α release	(64)
	HMB-PP	Vγ9Vδ2 TCR (proximal)	MEK/Erk + PI3K/Akt signal transduction	Vγ9Vδ2 T cell activation, proliferation, and cytotoxicity	(65)
	HMB-PP/pAgs	Vγ9Vδ2 TCR (proximal)	Calcium mobilization → NFAT nuclear translocation	Effector function activation; required for cytokine production	(66)
Co-stimulatory molecules	B7 (CD80/CD86) on APCs	CD28 on Vγ9Vδ2 T cells	Ig superfamily costimulatory pathway	IL-2 production; survival and proliferation	(71)
	CD70 on APCs	CD27 on Vγ9Vδ2 T cells	TNFR superfamily signaling	IFN-γ secretion; expansion upon pAg stimulation; γδ T cell differentiation	(72–74)
	CD30L on APCs	CD30 on activated Vγ9Vδ2 T cells	TNFR superfamily; enhanced calcium flux	Costimulation of IL-4, IFN-γ, and IL-8 production	(75, 76)
Direct TLR signaling	TLR2 agonists (e.g., PAM_2_CSK_4_)	TLR2 on Vγ9Vδ2 T cells	Synergizes with TCR signaling	Enhanced IFN-γ synthesis and secretion upon dual TCR+TLR2 stimulation	(77)
	TLR3 agonists (e.g., poly I:C)	TLR3 on Vγ9Vδ2 T cells	Integrated TCR+TLR3 signaling	Potent antiviral effector functions	(78, 79)
	TLR4 agonists (e.g., LPS)	TLR4 on Vγ9Vδ2 T cells	Synergizes with TCR signaling	Augmented IFN-γ release, proliferation, and cytotoxicity	(77, 80)
Indirect TLR signaling (via APCs)	TLR8 ligands (e.g., R848)	TLR8 on monocytes	Monocyte costimulation of γδ T cells	Early γδ T cell activation; IFN-γ production	(81)
	TLR2 ligands (BCG components)	TLR2 on DCs	DC maturation → IL-12p70 secretion → γδ T cell activation	Bidirectional γδ T–DC crosstalk; enhanced DC maturation and γδ T cell activation	(82)
	TLR3/TLR4 agonists	TLR3/TLR4 on DCs	Type I IFN production by DCs → Vγ9Vδ2 T cell activation	Rapid, robust IFN-γ production by Vγ9Vδ2 T cells (DC-dependent)	(83)
	TLR2/4 ligands (BCG) + memory CD4^+^ T cell-derived IFN-γ	TLR2/4 on DCs	DC-secreted IL-12p70 → indirect Vδ2^+^ γδ T cell activation	BCG-induced indirect activation of Vδ2^+^ T cells	(84)
NK receptor costimulation	MICA (on *Mtb*-infected DCs/epithelial cells)	NKG2D on Vγ9Vδ2 T cells	Augments TCR-dependent responses to pAgs	Enhanced TCR-dependent Vγ9Vδ2 T cell response to pAgs	(85)
	ULBP1 (stress-induced)	NKG2D on Vγ9Vδ2 T cells	NKG2D costimulatory signaling	Anti-infectious activity against intracellular bacteria	(86)
	NKG2D ligands (tumor/stress)	NKG2D on Vγ9Vδ2 T cells	PKCθ-dependent modulation of early TCR calcium signals	Costimulation of antitumor cytotoxicity; enhanced TNF-α and lytic granule release	(87, 88)
Cytokine signaling	IL-12	IL-12R on Vγ9Vδ2 T cells	PI3K/AKT + STAT4 activation; endogenous TNF-α signaling	Amplification of HMB-PP-activated Vγ9Vδ2 T cells; inhibition of intracellular mycobacterial growth (IFN-γ/TNF-α-dependent)	(67)
	IL-12 + IL-15 (synergistic)	IL-12R + IL-15R on Vγ9Vδ2 T cells	Dual STAT1 + STAT4 activation	Enhanced IFN-γ production by γδ T cells	(68)
	IL-2/IL-15	IL-2R/IL-15R on γδ thymocytes	Cytokine receptor signaling	Differentiation of γδ thymocytes into cytotoxic type 1 effectors (perforin^+^IFN-γ^+^)	(69)
Accessory signaling	Notch ligands (Jagged/Delta-like)	Notch1/Notch2 on γδ T cells	Notch signaling pathway	Facilitates antigen-specific effector functions; knockdown suppresses antitumor cytotoxicity	(70)

pAgs, phosphoantigens; TCR, T cell receptor; APC, antigen-presenting cell; PKCθ, protein kinase C theta; ERK, extracellular signal-regulated kinase; MAPK, mitogen-activated protein kinase; JNK, c-Jun N-terminal kinase; NF-κB, nuclear factor-kappa B; AP-1, activator protein-1; NFAT, nuclear factor of activated T cells; PI3K, phosphoinositide 3-kinase; AKT, protein kinase B; STAT, signal transducer and activator of transcription; MICA, MHC class I polypeptide-related sequence A; ULBP1, UL16-binding protein 1; NKG2D, natural killer group 2 member D; PAM_2_CSK_4_, palmitoyl tripeptide; LPS, lipopolysaccharide; DC, dendritic cell; IL, interleukin; IFN, interferon; TNF, tumor necrosis factor.

A key yet underappreciated aspect of TCR-dependent signaling is the potential for crosstalk and contradiction between parallel signaling pathways. For instance, while TCR engagement activates PKCθ and the MEK/Erk cascade (64, 65), IL-12 signaling through PI3K/AKT and STAT4 (67) may engage overlapping downstream nodes, raising the question of whether signal integration occurs cooperatively or competitively at key effector checkpoints. Moreover, the observation that Notch signaling is required for antitumor cytotoxicity (70) introduces an additional layer of complexity—whether Notch modulates TCR signal strength or operates through parallel transcriptional programs remains unclear. A contentious issue in the field concerns the signal integration logic governing the functional divergence of Vγ9Vδ2 T cells toward cytotoxic versus cytokine-secreting phenotypes: current models do not adequately explain how the same TCR stimulus, in the context of different cytokine milieus, can drive qualitatively distinct effector programs. The field lacks computational models that integrate multi-pathway signaling dynamics with functional outcome prediction for Vγ9Vδ2 T cells. In conventional αβ T cells, systems immunology approaches combining multiparameter phospho-flow cytometry with computational network inference have successfully mapped causal signaling relationships (89); applying similar frameworks to Vγ9Vδ2 T cell signaling represents an important next step.

### Co-stimulatory molecule signaling

Studies have demonstrated that activation of human and murine γδ T cells requires costimulation by CD28, a member of the immunoglobulin (Ig) superfamily; B7–CD28 interactions promote IL-2 production and govern γδ T cell survival and proliferation (71). However, in a murine Listeria infection model, γδ T cell differentiation into IL-17– and IFN-γ–producing cells contributes to bacterial clearance independently of CD28 costimulation (90). Concurrently, the majority of γδ T cells express CD27, a member of the tumor necrosis factor receptor (TNFR) superfamily; CD27 serves as a regulator of γδ T cell differentiation and IFN-γ secretion (72). The development of IFN-γ–producing γδ T cells appears to require costimulatory signals from both the TCR and CD27 in the thymus (73). De Barros et al. found that CD27 costimulatory signaling promotes the expansion and cytokine secretion of human Vγ9Vδ2 T cells upon TCR-mediated phosphoantigen stimulation (74). Following activation, human γδ T cells also express CD30, another TNFR superfamily member (75); CD30 exerts costimulatory effects in γδ T cells by enhancing intracellular calcium flux and augments the production of cytokines including IL-4, IFN-γ, and IL-8 (76).

### Direct TLR signaling in Vγ9Vδ2 T cells

TLRs can induce early rapid activation of Vγ9Vδ2 T cells and secretion of IFN-γ (83); dual stimulation of Vγ9Vδ2 T cells with anti-TCR antibodies and TLR2 agonists enhances IFN-γ synthesis and secretion, demonstrating the functional presence of TLR2 on Vγ9Vδ2 T cells (91). Concurrently, studies have revealed that γδ T cells express TLR3 (78) and TLR4 (77); integrated signaling via TLR3 and the TCR induces potent antiviral effector functions in γδ T cells (79), while TLR4 ligand (LPS) stimulation of γδ T cells augments IFN-γ release, cellular proliferation, and cytotoxic potential (80).

However, these findings regarding direct TLR signaling in Vγ9Vδ2 T cells are primarily derived from *in vitro* studies using purified cell systems or pharmacological TLR agonists, and their relevance to *in vivo* TB infection remains to be validated. A major concern is the supraphysiological concentrations of TLR ligands typically employed in these experiments, which may not reflect the local concentrations achievable within TB granulomas. Furthermore, the functional consequences of direct TLR signaling versus indirect TLR signaling (via APCs) *in vivo* may differ substantially: while direct TLR engagement on Vγ9Vδ2 T cells may provide rapid early activation, the indirect pathway through DC-derived IL-12 (82–84) likely sustains and shapes the effector response over time. The relative contributions of these two pathways at different stages of *Mtb* infection—early innate containment versus chronic adaptive immunity—have not been dissected experimentally. Additionally, conflicting reports exist regarding TLR expression levels across Vγ9Vδ2 T cell subsets and activation states (77, 78), suggesting that TLR responsiveness may be context-dependent and dynamically regulated, a complexity that has been insufficiently addressed in the literature.

### Indirect activation of Vγ9Vδ2 T cells via APC-mediated TLR signaling

Roelofs et al. identified monocytes as the cell type directly affected by bisphosphonates such as zoledronic acid, responsible for Vγ9Vδ2 T cell activation, and also as effective accessory cells for pAg-mediated activation of Vγ9Vδ2 T cells by HMB-PP and BrHPP (92); moreover, TLR8 ligand-activated monocytes efficiently costimulate early γδ T cell activation and induce IFN-γ production in γδ T cells (81). Niraj et al. (82) investigated the modulation of adaptive immune responses through interactions between human γδ T cells and dendritic cells (DCs) during infection with microorganisms such as *Mtb*, and found that TLR2 stimulation enhances γδ T cell activation, with activated γδ T cells subsequently promoting DC maturation and inducing secretion of cytokines including IL-12 p70. Devilder et al. (83) demonstrated that TLR3 and TLR4 agonists rapidly, robustly, and specifically induce IFN-γ production by Vγ9Vδ2 T cells, primarily dependent on type I IFNs produced by TLR-stimulated DCs. Another study explored the activation mechanisms of human Vδ2+ γδ T cells by the model pathogen BCG, revealing that Vδ2+ γδ T cells can be indirectly activated by BCG and DC-secreted IL-12p70, which is regulated by TLR2/4 ligands and IFN-γ secreted by memory CD4 T cells (84).

The bidirectional crosstalk between Vγ9Vδ2 T cells and APCs described above introduces an important consideration: the majority of studies examining APC–γδ T cell interactions have employed monocyte-derived DCs (moDCs) generated under artificial culture conditions, which may not faithfully recapitulate the properties of tissue-resident DC subsets in the lung mucosa. Recent evidence from spatial transcriptomic profiling of human TB granulomas has revealed remarkable macrophage and DC heterogeneity within these lesions (41), yet the specific interactions between these tissue-resident APC subsets and infiltrating Vγ9Vδ2 T cells remain entirely uncharacterized. The field lacks *in situ* evidence demonstrating that the APC–γδ T cell crosstalk observed *in vitro* occurs with similar efficiency within the hypoxic, nutrient-deprived, and immunosuppressive milieu of the TB granuloma.

### NK receptor costimulatory signaling

NKG2D, an activating receptor of NK cells, is recognized as a potent costimulatory receptor during antigen-specific activation of γδ and CD8 T cells; NKG2D enhances TNF-α production and lytic granule release in Vγ9Vδ2 T cells (87). Bessoles et al. emphasized the role of NKG2D and its ligands (e.g., ULBP1) in the anti-infectious activity of Vγ9Vδ2 T cells against intracellular bacteria (86). Stress-induced molecule MICA is upregulated on the surface of dendritic cells and epithelial cells upon *Mtb* infection, and its engagement with NKG2D augments TCR-dependent Vγ9Vδ2 T cell responses to pAgs (85). Additionally, NKG2D was found to costimulate antitumor cytotoxicity in human Vγ9Vδ2 T cells through PKCθ-dependent modulation of early TCR-induced calcium and transduction signals (88).

The redundancy within the Vγ9Vδ2 T cell activation network—encompassing TCR, TLR, NKG2D, Notch, and cytokine receptor signaling—raises fundamental questions about the hierarchy and compensatory mechanisms operating *in vivo*. If multiple pathways converge on similar effector outputs (IFN-γ production, cytotoxicity), what determines which pathway dominates under different infection contexts? This question has practical implications: therapeutic strategies targeting a single activation pathway (e.g., BTN3A1 agonistic antibodies) may be insufficient if compensatory upregulation of inhibitory pathways occurs. The concept of signal redundancy versus signal synergy in Vγ9Vδ2 T cell activation requires systematic investigation using combinatorial perturbation approaches, such as CRISPR-based screening of individual signaling components in the context of *Mtb*-infected macrophage–γδ T cell co-cultures.

## Vγ9Vδ2 T cell-mediated protective mechanisms against tuberculosis

**Figure 4:**
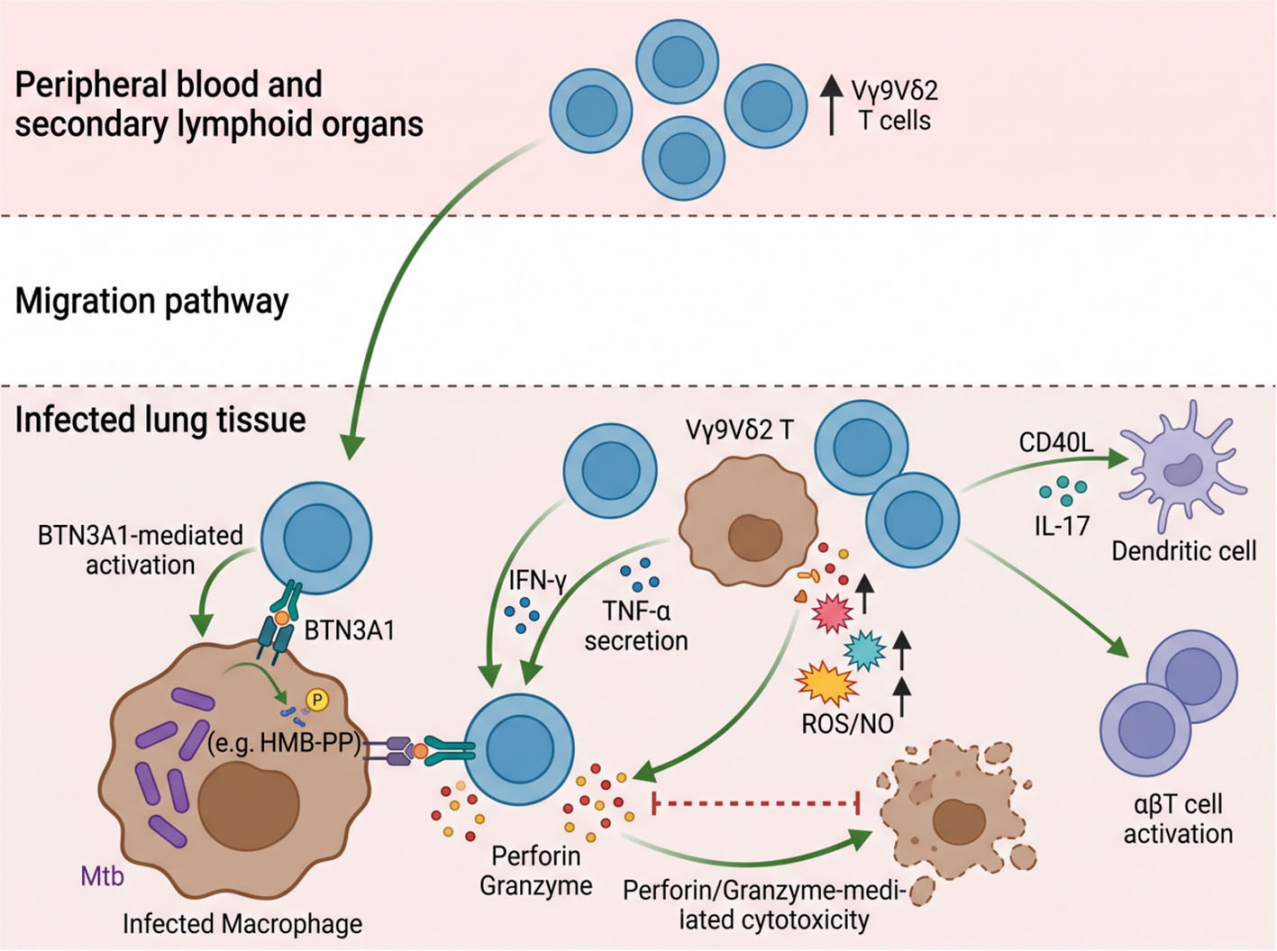
Vγ9Vδ2 T cell-mediated anti-tuberculosis protection. Multiple effector mechanisms contribute to Vγ9Vδ2 T cell-mediated protection against *Mycobacterium tuberculosis* (*Mtb*). Direct cytotoxicity via perforin/granzyme B (GZMB), Fas ligand (FasL)/Fas, and TNF-related apoptosis-inducing ligand (TRAIL) pathways. Cytokine-mediated immunoregulation, including interferon-γ (IFN-γ), tumor necrosis factor-α (TNF-α), and interleukin-17 (IL-17). Immunomodulation of dendritic cells (DCs), natural killer (NK) cells, and αβ T cells. *Mtb* immune evasion via metabolic antigen camouflage and functional exhaustion (programmed death-1 [PD-1], T cell immunoglobulin and mucin domain-containing protein-3 [TIM-3]). Assisted generation via jova.ai; edited, revised, and finalized by the authors.

The progression of TB is largely dependent on the capacity of *Mtb* to evade and exploit host immune responses, typically resulting in an equilibrium state characterized by immune containment and bacterial persistence (93) (**Figure 4**). *Mtb* evades host immune surveillance by inducing epigenetic alterations through reprogramming of host gene transcription, while simultaneously exploiting host macrophages and other immune cells to facilitate its evolution within the human host (94, 95). Evidence indicates that γδ T cells exert protective effects during infection and inflammation, particularly serving as important contributors to mycobacterium-specific type 1 immune responses—such as IFN-γ production and cytolytic activity—during *Mtb* infection, which are essential for tuberculosis immunity (96, 97). Vγ9Vδ2 T cells participate in the immune response to tuberculosis through multiple mechanisms, including antigen-specific expansion, direct killing of infected host cells, cytokine secretion, and modulation of other immune cell functions.

### Direct cytotoxic effects

During *Mtb* infection, the clonal expansion of Vγ9Vδ2 T cells represents an adaptive and antigen-specific response. Studies have demonstrated that γδT and MAIT cells undergo peripheral expansion during the acute phase of *Mtb* infection (98), with resident pulmonary lymphocytes expressing high levels of the γδ TCR (99), indicating their pivotal role at the frontline against tuberculosis infection. Flow cytometric detection of γδT cell expansion in exposed but uninfected contacts of TB patients supports the potential contribution of this population in responding to primary infection. Aerosol infection with H37Rv in non-human primates (NHPs) induced expansion of Vγ9Vδ2 T cells in the lung, accounting for up to 23% of CD3+ T cells (100). Acute active pulmonary tuberculosis is also associated with a reduction in circulating effector Vγ9Vδ2 T cells, while progressive depletion of Vδ2 T cells and production of suppressive cytokines correlate with the severity of pulmonary disease (101). Early studies found that γδ T cells are activated during primary immune responses to *Mycobacterium tuberculosis* or *Mycobacterium leprae*, and that γδ T cells respond to mycobacterial infection prior to αβ T cells, positioning them as early responders in the anti-mycobacterial immune cascade, alongside alveolar macrophages and other innate immune cells (102).

Vγ9Vδ2 T cells possess potent direct cytotoxic capabilities. Studies have demonstrated that the acquisition of cytotoxic potential by Vδ2 T cells during postnatal development is exquisitely regulated, wherein expression levels of PD-1 and CD56 are closely associated with perforin production, and the transcription factor IRF8 may play a pivotal role in this differentiation process (103). In tuberculosis, Vγ9Vδ2 T cells can recognize and respond to phosphoantigens produced by *Mtb*-infected cells, rapidly activating and proliferating to eliminate infected host cells through the release of effector molecules such as perforin and granzyme B, thereby inducing apoptosis of infected cells (104). Notably, Dieli et al. demonstrated that Vγ9Vδ2 T cells can directly kill both extracellular and intracellular *Mtb* through a granule-dependent mechanism involving granulysin and perforin. Granulysin was identified as the pivotal mycobactericidal molecule, as depletion of granulysin from Vγ9Vδ2 T cell supernatants abrogated both intracellular and extracellular *Mtb* killing, whereas perforin was required for killing of infected macrophages but did not possess direct microbicidal activity (105). Vγ9Vδ2 T cells can also induce target cell apoptosis through expression of Fas ligand (FasL) and TNF-related apoptosis-inducing ligand (TRAIL). Engagement of FasL with Fas receptor on target cells, or binding of TRAIL to death receptors (e.g., DR4/DR5), initiates the extrinsic apoptotic pathway, leading to clearance of infected cells (106). This mechanism facilitates elimination of infected cells while minimizing excessive inflammatory damage due to the absence of robust proinflammatory cytokine release, thereby maintaining immune homeostasis. Within tuberculous granulomas, this apoptosis-inducing function may contribute to the clearance of senescent or dysfunctional immune cells, promoting granuloma homeostasis.

While the Dieli et al. study (105) identifying granulysin as the pivotal mycobactericidal molecule has been widely cited, these findings have not been robustly replicated by independent groups using distinct experimental systems. The original study employed a relatively small number of donors and a specific *in vitro* killing assay, and subsequent investigations have yielded a more nuanced picture. While the mycobactericidal activity of granulysin has been confirmed in multiple experimental systems (107, 108) and correlated with protective immune responses in TB patient cohorts (109), recent studies indicate that no single cytotoxic molecule is sufficient to account for intracellular *Mtb* killing; rather, the combined action of perforin, granulysin, and granzyme B—so-called polycytotoxic T cells (P-CTLs)—is required (110). This unresolved controversy is significant because granulysin represents a unique effector mechanism specific to cytotoxic lymphocytes; if its contribution to anti-TB immunity is less universal than originally proposed, therapeutic strategies aimed at enhancing granulysin production may have limited clinical impact. Furthermore, the majority of cytotoxicity studies have been conducted using short-term *in vitro* co-culture systems that do not recapitulate the complex three-dimensional architecture of TB granulomas, where oxygen tension, nutrient availability, and cell-cell contact patterns may fundamentally alter effector molecule accessibility to intracellular *Mtb*. Recent single-cell RNA sequencing studies of γδ T cells from TB patient peripheral blood and pleural effusion have revealed substantial functional heterogeneity among cytotoxic subsets (111), suggesting that the relative contributions of perforin/granzyme versus granulysin may vary across anatomical compartments and disease stages—a complexity that has not been adequately addressed in the existing literature.

*Mtb*-specific Vγ9Vδ2 T cells were detected in macaques through TCR CDR3-specificity analysis, demonstrating differential distribution across distinct tissues (lymphatic system, lung, kidney, and liver) associated with infection stage; Vγ9Vδ2 T cells derived from tissue compartments were capable of exerting effector functions and producing anti-mycobacterial cytokines (112). Using anti-δ chain antibodies to examine patients with pulmonary tuberculosis, bacterial pneumonia, or other lower respiratory tract infections, increased γδ T cells were observed in the blood of confirmed HIV-negative pulmonary tuberculosis patients. The anti-TB activity of γδ T cells and their MHC-unrestricted nature render their recognition of highly conserved antigens an attractive vaccine target; concurrently, TB-specific γδ T cells exhibit a pronounced propensity to migrate to and reside within lung tissue, conferring protective activity upon γδ T cells, with T cell control potentially varying across different lesions within the same lung (39).

### Immunomodulatory functions

In addition to their direct cytotoxic functions,Vγ9Vδ2 T cells secrete diverse cytokines, including interferon-γ (IFN-γ) and tumor necrosis factor-α(TNF-α) (113), which enhance the bactericidal capacity of macrophages and inhibit *Mtb* replication. Vγ9Vδ2 T cells express natural killer (NK) cell receptors such as NKG2D, and through recognition of stress-induced molecules on the surface of infected cells, further augment their cytotoxic effects (87). Concurrently,Vγ9Vδ2 T cells are capable of secreting interleukin-17 (IL-17), promoting neutrophil recruitment and activation (114), which is essential for early containment of *Mtb* infection.

Vγ9Vδ2 T cells also participate in modulating the activity of other immune cells. Through interactions with dendritic cells (DCs), they promote DC maturation and functionality, thereby enhancing antigen presentation capacity and initiating adaptive immune responses against mycobacteria by CD4+ and CD8+ T cells. It should be noted that this finding was established in a bovine model using *Mycobacterium bovis*-infected dendritic cells (115), which, while informative, may not fully recapitulate the dynamics of *Mtb* infection in human systems. Upon stimulation with *Mtb* antigens, activated NK cells improve γδT cell proliferation via immunological synapse formation and cell–cell contact mediated by soluble factors including TNF-α, GM-CSF, and IL-12, indicating an interplay between NK cells and γδT cells in anti-tuberculosis immunity (116). The *Mtb* antigen ESAT-6 can activate and induce proliferation of γδT cells; concurrently, regulatory T cells (Tregs) have been found to suppress IFN-γ production by human memory γδT cells in response to the *Mtb* antigen ESAT-6 (117).Vγ9Vδ2 T cells can also enhance the expansion of *Mtb*-specific αβ T cells and B cells and augment their capacity to inhibit intracellular mycobacteria, exerting effects analogous to those of CD4^+^ αβ T cells in immunoenhancement and antigen presentation by *Mtb*-specific T cells (19); moreover, the antimicrobial effects of Vγ9Vδ2 T cells depend not only on soluble factors but also on intercellular contact involving CD40–CD40 ligand interactions (118). Furthermore, Vγ9Vδ2 T cells possess immunoregulatory functions, such as promoting tissue repair through IL-22 secretion, or coordinating the overall immune response network through interactions with other immune cells including NK cells and αβ T cells (119).

Despite the breadth of immunomodulatory functions attributed to Vγ9Vδ2 T cells, several important limitations in the current evidence warrant acknowledgment. First, the majority of studies demonstrating DC maturation (115), NK cell crosstalk (116), Treg-mediated suppression (117), and CD40/CD40L-dependent antigen presentation (118) were conducted using *in vitro* co-culture systems with supraphysiological effector-to-target ratios or ex vivo phosphoantigen stimulation; whether these interactions occur at comparable magnitude during natural Mtb infection *in vivo* remains unestablished. Second, the relative contribution of each immunomodulatory pathway—cytokine-mediated macrophage activation versus direct cytotoxicity versus DC licensing—to actual TB protection has not been quantitatively dissected, making it impossible to determine which effector axis is rate-limiting for bacterial control. Third, the temporal dynamics of immunomodulatory function are poorly characterized: it is unknown whether the immunoregulatory properties of Vγ9Vδ2 T cells (e.g., IL-22-mediated tissue repair) are maintained, diminished, or functionally reprogrammed during the transition from acute infection to chronic disease. These gaps underscore the need for *in vivo* models and longitudinal clinical studies that can establish causal, rather than correlative, relationships between Vγ9Vδ2 T cell immunomodulatory activities and TB outcomes.

Notably, phosphoantigen-reactive γδ T cells expanded during acute *Mtb* infection are Vδ2 T cells, whereas during chronic *Mtb* infection, expansion occurs in a distinct NK-like CD8+ γδ T cell subset (predominantly Vδ1) (6) This raises the question of whether circulating Vδ2 T cells can infiltrate infected lung tissue to eliminate *Mtb*; further elucidation of the functional diversity among γδ T cell subsets and their mediated TB immune response mechanisms may contribute to the development of γδ T cell-based immunotherapy. Li et al. (120) has also identified the *Mtb* protein Rv0002 as a potential ligand capable of binding to the γδ TCR, thereby activating γδ T cell-mediated immunity.

This paradoxical Vδ2→Vδ1 functional shift during the transition from acute to chronic *Mtb* infection represents one of the most contentious issues in the field. The observation that chronic *Mtb* infection is associated with NK-like CD8+ Vδ1 T cell expansion (6), while Vδ2 T cells become depleted from circulation (20, 101), raises several unresolved questions. First, is the Vδ2→Vδ1 shift a cause or consequence of disease progression? The available cross-sectional data cannot distinguish between these possibilities, and longitudinal studies tracking individual patients from latent infection through active TB development are conspicuously absent. Second, the mechanism driving this subset switch remains unclear—does it reflect differential tissue tropism, selective exhaustion and deletion of Vδ2 cells in the periphery, or active *Mtb*-driven suppression of the Vδ2 compartment? Third, from a translational perspective, if Vδ1 T cells predominate at chronic infection sites but Vγ9Vδ2 T cells are the target of most therapeutic strategies, this creates a fundamental mismatch between the intended therapeutic agent and the cells actually present at the disease site. Recent single-cell transcriptomic analyses have begun to address this heterogeneity, revealing that Vδ2 T cells from active TB patients exhibit distinct transcriptional programs compared to healthy donors, with downregulation of cytotoxic pathways and upregulation of inflammatory and apoptotic signatures (111). However, these studies remain limited by small sample sizes and the absence of paired longitudinal samples, limiting their ability to establish causal relationships between subset dynamics and disease outcomes.

### *Mtb* immune evasion and Vγ9Vδ2 T cell functional exhaustion

The evasion mechanisms of *Mtb* and immune exhaustion resulting from chronic infection constitute key factors influencing Vγ9Vδ2 T cell functionality. *Mtb* can modulate host cellular metabolic pathways to reduce phosphoantigen production, thereby evading recognition by Vγ9Vδ2 T cells. HMB-PP, an intermediate metabolite of the isoprenoid biosynthesis pathway in *Mtb*, serves as the principal phosphoantigen activating Vγ9Vδ2 T cells. Studies have demonstrated that under specific microenvironmental stressors, such as hypoxia or nutrient restriction, *Mtb* adjusts its metabolic network to diminish HMB-PP synthesis and release, consequently attenuating Vγ9Vδ2 T cell perception through the TCR (121). This metabolic “antigen camouflage” enables *Mtb* to evade rapid γδ T cell surveillance during the early stages of infection.

Furthermore, chronic *Mtb* infection may induce functional exhaustion of Vγ9Vδ2 T cells, attenuating their immunological competence (122). In the context of HIV/*Mtb* coinfection, this immune evasion effect becomes more complex, as HIV infection per se leads to decreased frequency and functionality of the Vδ2 T cell subset, further compromising host control over *Mtb* (123). In diabetes mellitus (DM/prediabetes, PDM) comorbid with latent tuberculosis (LTB), γδ T cells exhibit comprehensive impairment of anti-*Mtb* effector functions characterized by reduced secretion of Th1 cytokines (IFN-γ, TNF-α) and Th17 cytokines (IL-17 family), diminished expression of cytotoxic molecules (perforin, granzyme B, granulysin), and decreased frequencies of the immune checkpoint receptor PD-1 and the activation marker CD69. Consequently, these cells fail to effectively activate and regulate other immune cells or directly kill infected cells, resulting in attenuated immune protection that facilitates *Mtb* immune evasion and disease progression (124).

Alterations in Vγ9Vδ2 T cell phenotype and/or function have been reported in infections caused by intracellular pathogens. The absence of CD26^+^CD161^+^TRAV1-2^-^IFN-γ^+^CD4^+^ T cell effectors—distinct from MAIT cells, which express TRAV1-2—in immunocompromised hosts and during active pulmonary tuberculosis leads to reduced frequencies of IFN-γ–producing cells following stimulation with non-peptide mycobacterial ligands (125). A phenotypic and functional analysis of Vγ9Vδ2 T cells in blood and bronchoalveolar lavage fluid from tuberculosis patients revealed that increased blood Vγ9Vδ2 T cells displayed potential cytotoxic activity; however, pulmonary Vγ9Vδ2 T cells exhibited significant downregulation of CD3 molecules and markedly impaired responsiveness to antigen stimulation, with incapacity for cytokine production (IFN-γ, TNF-α) (126). These findings suggest that high bacterial burden induces chronic stimulation of effector Vγ9Vδ2 T cells, potentially resulting in their deletion or exhaustion. Indeed, anergic pulmonary tuberculosis is accompanied by decreased percentages of Vδ2^+^ T cells, elevated FasL expression on Vδ2^+^ T cells, and enhanced peripheral blood levels of IL-4 and IL-10, which induce Vδ2^+^ T cell–mediated impairment of anti-tuberculosis immunity, accounting for the severe clinical tuberculosis symptoms observed in anergic pulmonary tuberculosis patients (101). Therefore, a comprehensive understanding of *Mtb* evasion strategies targeting Vγ9Vδ2 T cells holds significant implications for the development of novel anti-tuberculosis immunotherapies and vaccines.

Despite the recognition of metabolic antigen camouflage as a key *Mtb* evasion strategy, the mechanistic details remain largely speculative. A major unresolved question is how *Mtb* precisely calibrates HMB-PP production in response to host microenvironmental cues. The MEP pathway is subject to complex feedback regulation, and the specific metabolic checkpoints at which *Mtb* downregulates HMB-PP under hypoxic or nutrient-limited conditions have not been identified at the molecular level. Furthermore, while it has been established that anergic TB is associated with Vδ2 T cell contraction (101), the epigenetic mechanisms underlying this functional impairment remain poorly characterized. Emerging evidence from epigenetic profiling of T cells in chronic infections suggests that sustained antigen exposure drives progressive chromatin remodeling at key effector gene loci, culminating in a terminally exhausted state marked by DNA methylation of the IFNG locus and enhancer regions (127). Whether analogous epigenetic reprogramming occurs in Vγ9Vδ2 T cells during chronic *Mtb* infection—potentially involving histone modifications at the BTN3A1 locus that silence its expression and further blunt phosphoantigen sensing—represents an unexplored frontier. Additionally, while comorbidity-associated functional impairment (HIV, diabetes) has been documented (123, 124), the field lacks integrated multi-omics studies that simultaneously profile transcriptional, epigenetic, and metabolic changes in Vγ9Vδ2 T cells across the spectrum of TB disease states, from latent infection through active disease to post-treatment recovery.

## Vγ9Vδ2 T cell immunological memory and vaccine strategies

### Characteristics of immunological memory

Vγ9Vδ2 T cells may acquire an effector memory T cell phenotype (tissue-resident effector memory, TEM cells) following multiple infections, a characteristic reported in bacterial, parasitic, and viral infections (26, 128). BCG vaccination enhances human γδ T cell responses to *Mtb* and fosters the development of a memory immune-like phenotypic population (129). Further studies have revealed that primary BCG vaccination in infants elevates γδ T cell frequencies, suggesting their involvement as early responder cells in vaccine-induced immunity, while concurrently inducing increased frequencies of a novel CD26^+^CD161^+^TRAV1-2^-^IFN-γ^+^CD4^+^ T cell subset (distinct from MAIT cells, which are defined by TRAV1–2 expression), which may represent a novel candidate population for tuberculosis protective immunity (130). In a BCG-infected macaque model, phosphoantigen-specific Vγ9Vδ2 T cells underwent substantial expansion and exhibited a pronounced memory-type response 4 to 6 days following BCG reinfection, with expansion magnitudes 2- to 9-fold greater than those observed during primary infection (131). Shen et al. further demonstrated that early expansion and differentiation of Vγ2Vδ2 T cells in an *Mtb*-infected NHP model enhances macaque resistance to TB, eliciting rapid-acting and durable memory-like responses, suggesting that immune Vγ9Vδ2 T cells may represent a promising strategy for tuberculosis vaccine development (132).

**Figure 5:**
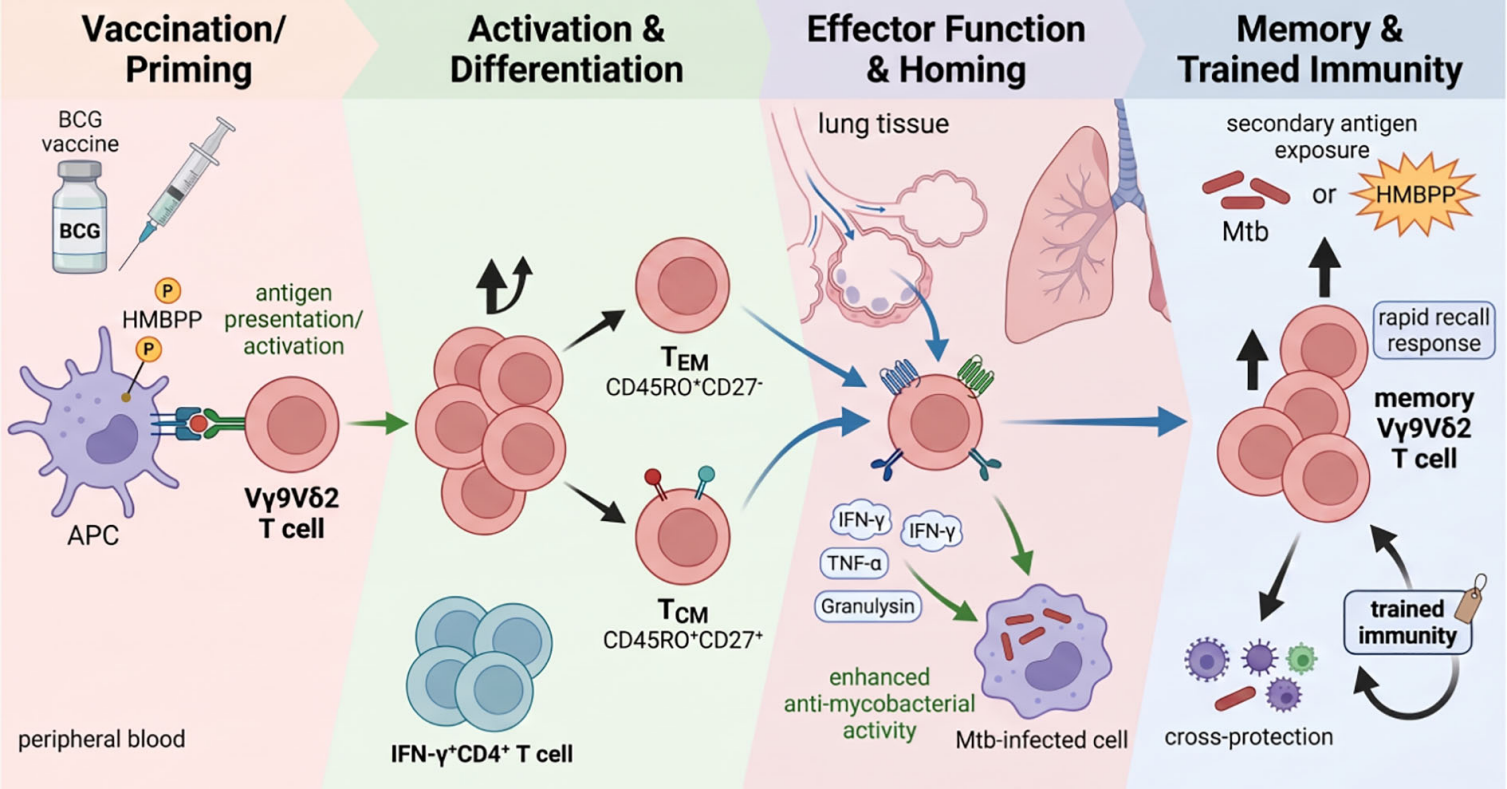
BCG vaccination-induced Vγ9Vδ2 T cell immune memory. Intravenous (IV) bacillus Calmette-Guérin (BCG) induces substantial Vδ2 T cell expansion associated with effector memory (EM)/central memory (CM) phenotypes and protective efficacy. Aerosol BCG booster enables pulmonary localization of activated Vδ2 T cells. BCG also reprograms γδ T cell transcriptional programs to develop trained immunity. IFN-γ, interferon-γ; TNF-α, tumor necrosis factor-α; IL-2, interleukin-2. Assisted generation via jova.ai; edited, revised, and finalized by the authors.

In non-human primates, intravenous BCG vaccination induces substantial expansion of the Vδ2 T cell population, increasing from a median of 1.5% of CD3^+^ cells at baseline to 5.3% at 4 weeks post-*Mtb* challenge, with this expansion closely associated with tuberculosis protective efficacy. This protective effect correlates with effector memory and central memory phenotypes, homing markers, and production of IFN-γ, TNF-α, and granulysin (133). In contrast, intradermal BCG administration fails to induce Vδ2 T cell expansion and elicits only limited phenotypic and functional alterations, indicating that the route of vaccine delivery is essential for the induction of Vγ9Vδ2 T cell memory (133). Furthermore, aerosol BCG booster immunization enables localization of activated Vδ2 T cell populations at pulmonary mucosal surfaces, suggesting that this strategy may overcome the limitation of intradermal BCG-induced immune responses in migrating from the circulatory system to the site of infection (133). These findings underscore the advantages of phosphoantigen-based vaccine strategies in inducing Vγ9Vδ2 T cell memory. A recent study integrating single-cell sequencing with immunological functional analysis revealed that BCG reprograms γδ T cell transcriptional programs, enhancing their reactivity to heterologous stimuli such as bacteria and fungi, and augmenting TNF and IFN-γ secretion for several weeks, suggesting that human γδ T cells can be induced by BCG to develop a trained immunity phenotype, contributing to the heterologous protective effects of BCG (134) (**Figure 5**).

The concept of “trained immunity” in γδ T cells, as proposed by Suen et al. (134), is conceptually contentious. Trained immunity—originally defined as enhanced innate immune responsiveness following exposure to microbial stimuli, mediated by metabolic and epigenetic reprogramming—has been well-established in monocytes, macrophages, and NK cells, but its extension to γδ T cells is conceptually contentious. The fundamental debate centers on whether the observed enhanced responsiveness of BCG-exposed γδ T cells represents genuine “memory” (which implies antigen-specific reactivation with clonal expansion) or innate reprogramming (a non-specific enhancement of responsiveness to heterologous stimuli). Unlike canonical T cell memory, which is characterized by clonal expansion, long-lived survival, and accelerated secondary responses specific to the cognate antigen, the BCG-induced γδ T cell “trained immunity” phenotype described by Suen et al. features enhanced reactivity to diverse stimuli (bacteria, fungi) without evidence of antigen specificity. This observation sits in tension with the classical immunological definition of memory and raises the question of whether the term “trained immunity” rather than “immunological memory” more accurately describes this phenomenon. Resolution of this debate has significant implications for vaccine design: if γδ T cells develop antigen-specific memory analogous to αβ T cells, vaccines should be optimized to maximize phosphoantigen-specific clonal expansion; if they instead undergo innate reprogramming, the optimal strategy would focus on non-specific immune enhancement. Notably, recent studies using ATAC-seq have begun to characterize the epigenetic landscape of trained γδ T cells in a bovine BCG model, revealing altered chromatin accessibility at innate immunity-related gene loci that parallel features of trained immunity observed in myeloid cells (135); however, definitive proof of the epigenetic mechanism in Vγ9Vδ2 T cells specifically remains lacking.

A important limitation of current vaccine studies targeting Vγ9Vδ2 T cell memory is the near-exclusive reliance on NHP models. While NHPs faithfully recapitulate many aspects of human TB pathogenesis—including granuloma formation, latent infection, and reactivation—they differ from humans in several immunological parameters that may affect Vγ9Vδ2 T cell responses, including differences in γδ T cell frequency, tissue distribution, and TCR repertoire diversity. Moreover, the controlled infection conditions in NHP studies (defined inoculum, uniform genetic background, specific pathogen-free status) do not reflect the heterogeneous exposure conditions, comorbidities, and genetic diversity of human populations. The field would benefit from complementary studies in humanized mouse models (17) and more importantly from clinical correlation studies that validate NHP-derived correlates of protection in human cohorts with defined TB outcomes.

### Vγ9Vδ2 T cell-based vaccine design strategies

Future vaccine optimization strategies should focus on enhancing the memory-inducing capacity of BCG toward Vγ9Vδ2 T cells (129). These strategies can be broadly categorized into four approaches: optimization of existing vaccine platforms, development of Vγ9Vδ2 T cell-selective vaccines, engineering of recombinant BCG with enhanced phosphoantigen production, and incorporation of non-phosphoantigen mycobacterial antigens (**Table 2**).

**Table 2:** Vaccine strategies targeting Vγ9Vδ2 T cells.

#	Vaccine strategy	Key antigen/component	Vγ9Vδ2 T cell response	Model system	Key findings	Reference
1	BCG (intravenous route)	Live attenuated *M. bovis* BCG (IV delivery)	γδ T cell expansion with EM/CM phenotype; superior to intradermal route	NHP	IV BCG vaccination induced strongest γδ T cell responses; associated with TB protective efficacy against *Mtb* challenge	(133)
2	BCG (aerosol revaccination)	Live attenuated *M. bovis* BCG (aerosol delivery)	Pulmonary γδ T cell localization and activation	NHP	Enhanced pulmonary γδ T cell responses vs intradermal route; however, protective efficacy inferior to IV BCG	(133)
3	MTBVAC	Live attenuated *Mtb* (ΔphoP/ΔfadD26)	CD16^+^GZMB^+^GNLY^+^CX3CR1^+^ cytolytic γδ T cell expansion (~40% CD16^+^ vs ~20% for BCG); CD25^+^HLA-DR^+^CXCR3^+^ activation; no PD-1 upregulation	*In vitro* (human PBMCs, n=11)	Early cytolytic phenotype without exhaustion markers; distinct from BCG-expanded γδ T cells	(22)
4	BCG reinfection memory response	Endogenous phosphoantigen from BCG reinfection	Memory-type Vγ2Vδ2 T cell expansion (2–9 fold greater than primary response), EM/CM phenotype	NHP	First demonstration of Vγ2Vδ2 T cell adaptive memory response; recall expansion at 4–6 days post-reinfection	(131)
5	HMBPP-producing *L. monocytogenes*	Attenuated LmΔactA/prfA* (respiratory vaccination); ΔgcpE strain as HMBPP-negative control	Th1-like IFN-γ^+^perforin^+^ tissue-resident Vγ2Vδ2 effectors in airways and lungs; prolonged expansion ≥3 months	NHP (rhesus macaques)	Reduced lung pathology and extrapulmonary dissemination after *Mtb* challenge; ΔgcpE control ineffective, confirming HMBPP dependence; enhanced CD4^+^/CD8^+^ pulmonary Th1 responses	(132)
6	Recombinant BCG-gcpE	BCG overexpressing HMBPP synthase GcpE (pCQ105)	Potently enhanced Vγ9Vδ2 T cell activation over WT BCG ex vivo	*In vitro* (human PBMCs)	GcpE overexpression circumvented MEP pathway feedback inhibition; no bacterial growth defect; synthetic multi-gene MEP locus failed due to feedback inhibition	(58)
7	mGLP-based vaccine	6-O-Methylglucose lipopolysaccharide (mGLP, mycobacterial glycolipid)	BTN3A1-dependent but phosphoantigen-independent protective Vγ9Vδ2 subset activation	*In vitro*	Activated protective Vγ9Vδ2 subset that inhibited intracellular *Mtb* growth; acylated glucosyl residues at non-reducing terminus essential for activation	(60)
8	ProPAgens (phosphoantigen prodrugs)	Aryloxy diester phosphonamidate prodrugs (P-ProPAgens); acetate-protected double prodrugs	Enhanced Vγ9Vδ2 T cell proliferation and cytokine production exceeding natural phosphoantigens	*In vitro*	Improved drug-like properties (cellular permeability, metabolic stability); higher γδ T cell activation activity than natural pAgs	(136, 137)

EM, effector memory; CM, central memory; GZMB, granzyme B; GNLY, granulysin; NHP, non-human primate; MDR-TB, multidrug-resistant tuberculosis; ZOL, zoledronate; rIL-2, recombinant interleukin-2; IV, intravenous; PBMC, peripheral blood mononuclear cell; HLA-DR, human leukocyte antigen-DR; CXCR3, C-X-C chemokine receptor type 3; PD-1, programmed death-1; HMBPP, (E)-4-hydroxy-3-methyl-but-2-enyl pyrophosphate; Lm, Listeria monocytogenes; mGLP, 6-O-methylglucose lipopolysaccharide; BTN3A1, butyrophilin 3A1; ProPAgens, phosphoantigen prodrugs; pAgs, phosphoantigens; WT, wild-type; MEP, methylerythritol phosphate.

### Optimization of existing vaccine platforms

Improving vaccine delivery routes represents an important direction. Intravenous or aerosol routes, compared with conventional intradermal injection, more effectively induce Vγ9Vδ2 T cell expansion and pulmonary tissue localization, which is particularly important for *Mtb*, a pathogen transmitted via the respiratory tract (134). In a comparative NHP study, IV BCG vaccination induced the strongest γδ T cell responses and was associated with protective efficacy against *Mtb* challenge, whereas aerosol BCG revaccination enhanced pulmonary γδ T cell localization but was inferior to the IV route (134). Adjuvant combination can augment Vγ9Vδ2 T cell activation signals. For instance, TLR agonists can indirectly enhance γδ T cell responses through DC-mediated IL-12 production, while synergistic IL-12 and IL-15 signaling further promotes IFN-γ production (67, 68).

Notably, MTBVAC, a live attenuated *Mtb* strain with deletions in phoP and fadD26 (genes essential for virulence lipid synthesis), has advanced to Phase III clinical trials and exhibits a distinctive Vγ9Vδ2 T cell activation profile. *In vitro* studies comparing MTBVAC- and BCG-stimulated human PBMCs revealed that MTBVAC induces expansion of CD16^+^GZMB^+^GNLY^+^CX3CR1^+^ cytolytic γδ T cells at approximately twice the frequency of BCG (~40% vs ~20% CD16^+^), with concurrent upregulation of CD25, HLA-DR, and CXCR3, and notably, without PD-1 upregulation (22). This early cytolytic phenotype without exhaustion markers contrasts with the PD-1^+^ phenotype observed following aminobisphosphonate-mediated Vδ2 T cell expansion, suggesting that *MTB*VAC may preserve γδ T cell functionality more effectively than pharmacologic Vγ9Vδ2 T cell activation strategies.

However, the MTBVAC-induced Vγ9Vδ2 T cell activation data derive exclusively from *in vitro* PBMC stimulation assays (22), and whether this favorable phenotype is maintained *in vivo* during natural mucosal infection remains unknown. The translation from *in vitro* stimulation to *in vivo* protective efficacy represents an important gap that can only be addressed through the ongoing Phase III clinical trials with integrated immunological monitoring.

### Vγ9Vδ2 T cell-selective vaccination

The most compelling preclinical evidence for Vγ9Vδ2 T cell-selective vaccination comes from an NHP study employing respiratory administration of an HMBPP-producing attenuated *Listeria monocytogenes* strain (LmΔactA/prfA). A single respiratory vaccination of macaques with LmΔactA/prfA caused prolonged expansion of HMBPP-specific Vγ2Vδ2 T cells in both circulating and pulmonary compartments, eliciting Th1-like Vγ2Vδ2 T cell responses and reducing TB infection and pathology after moderate-dose *Mtb* challenge. Crucially, immunization with an HMBPP-deficient ΔgcpE Listeria strain failed to expand Vγ2Vδ2 T cells or confer protection, confirming that the protective effect was HMBPP-dependent (132). Vaccine effects coincided with tissue-resident Vγ2Vδ2 effector T cells coproducing IFN-γ and perforin and inhibiting intracellular *Mtb* growth, as well as earlier pulmonary CD4^+^ and CD8^+^ Th1 responses, suggesting that selective Vγ9Vδ2 T cell immunization can amplify responses of other T cell subsets (132). These findings establish a concept of immunizing human Vγ9Vδ2 T cells for TB vaccine development.

### Engineering recombinant BCG with enhanced HMBPP production

An alternative strategy to enhance Vγ9Vδ2 T cell responses involves engineering BCG to overproduce HMBPP via synthetic biology approaches. Qabar et al. (58) leveraged synteny analyses across >63 mycobacterial species to design synthetic MEP pathway loci for expression in BCG. While a multi-gene synthetic MEP locus failed to enhance Vγ9Vδ2 T cell expansion over wild-type BCG due to feedback inhibition of the pathway, overexpression of the HMBPP synthase GcpE alone potently induced Vγ9Vδ2 T cell expansion without concomitant downregulation of other pathway genes or bacterial growth defects. This targeted strategy circumvents the tight metabolic regulation of the MEP pathway and presents a promising system to improve upon the BCG platform, although *in vivo* validation in animal models is still needed.

### Non-phosphoantigen mycobacterial antigens as vaccine components

In addition to phosphoantigens, Vγ9Vδ2 T cells can recognize certain non-phosphorylated antigens through distinct recognition pathways (**Table 2**). In the context of infected cells, potent mitogens such as staphylococcal enterotoxin A (59) can activate γδ T cells; however, unlike classical superantigens that bind MHC class II molecules and polyclonally activate αβ T cells, these molecules engage γδ T cells through distinct, MHC-independent mechanisms. Several studies have characterized novel mycobacterial protein antigens recognized by human γδ TCR-bearing cells, including the oxidative stress response regulatory protein (OxyS) (57), 1-deoxy-D-xylulose 5-phosphate synthase 2 (DXS2), and the Rv2272 extracellular peptide (EP), which can activate γδ T cells from BCG-vaccinated human subjects and mediate cytotoxic effects against mycobacteria. These protein/peptide antigens directly engage the γδ TCR independently of BTN molecules, representing a fundamentally distinct recognition pathway from phosphoantigen/BTN-mediated activation. Xia et al. employed mass spectrometry analysis and immunological imaging techniques to investigate tuberculosis-associated antigens capable of inducing protective Vγ9Vδ2 T cell expansion; specifically, 6-O-methylglucose lipopolysaccharide (mGLP), a polar glycolipid component of the mycobacterial cell wall, was found to stimulate enhanced activity of a BTN3A1-dependent but phosphoantigen-independent protective subset of Vγ9Vδ2 T cells and effectively inhibit intracellular *Mtb* growth (60), potentially offering novel immunotherapeutic strategies for both drug-susceptible and drug-resistant tuberculosis. The *Mtb* cell wall is enriched in lipids, and these lipid antigens can be recognized by γδ T cells. For instance, *Mycobacterium tuberculosis* phosphomycoketide represents a lipid molecule presented by CD1c to Vδ1 TCR-expressing γδ T cells (61), constituting yet another independent antigen recognition pathway. Consequently, mGLP and mycobacterial ketides appear to serve as specific antigens suitable for incorporation into tuberculosis vaccines.

### Phosphoantigen prodrugs for vaccine applications

The development of novel vaccines based on phosphoantigens constitutes an important strategy for directly targeting Vγ9Vδ2 T cells. HMB-PP, the most potent natural phosphoantigen, and its analogues or prodrug forms (ProPAgens) with improved drug-like properties, may serve as vaccine components for direct activation of Vγ9Vδ2 T cells (136, 137). However, the instability and polar nature of the pyrophosphate moiety in natural phosphoantigens constrain their direct clinical application. ProPAgens, including aryloxy diester phosphonamidate prodrugs and acetate-protected double prodrugs, exhibit higher Vγ9Vδ2 T cell proliferation and cytokine production activity than natural phosphoantigens while offering improved cellular permeability and metabolic stability (136, 137).

While phosphoantigen prodrugs represent a rational approach to overcome the pharmacological limitations of natural pAgs, their application as vaccine components faces unresolved challenges. The optimal delivery system for phosphoantigen prodrugs in a vaccine context—whether incorporated into lipid nanoparticles, conjugated to carrier proteins, or encapsulated in slow-release formulations—has not been systematically evaluated. Moreover, the potential for repeated phosphoantigen administration to induce Vγ9Vδ2 T cell exhaustion rather than memory remains a concern, as chronic pAg exposure in the context of active TB is associated with functional impairment (101, 126). The development of controlled-release delivery systems that provide pulsed antigen exposure, mimicking the natural kinetics of infection-resolution cycles, may be essential to optimize memory induction while minimizing exhaustion risk—a concept that has not yet been explored experimentally.

### Neonatal vaccination window

Human Vγ9Vδ2 T cells of fetal TCR repertoire origin expand within 10 weeks postpartum, exhibiting potent cytotoxic functionality as a protective mechanism against sudden environmental microbial phosphoantigen exposure, such as neonatal *Mycobacterium tuberculosis* (34). This finding suggests that optimizing BCG vaccination strategies during the neonatal period, thereby fully exploiting the innate protective potential of Vγ9Vδ2 T cells, may represent a key window for improving vaccine protective efficacy. The observation that IV BCG more potently activates γδ T cells than intradermal BCG in NHP models (133) further supports the investigation of alternative delivery routes during this neonatal window.

However, the neonatal vaccination window strategy raises important safety and practical concerns that have not been adequately addressed. First, while fetal-derived Vγ9Vδ2 T cells exhibit potent cytotoxicity (34), the ontogeny of functional BTN3A1/BTN2A1 expression in neonatal antigen-presenting cells has not been systematically characterized—if the “inside-out” antigen sensing machinery is immature at birth, the functional advantage of early vaccination may be diminished. Second, in TB-endemic settings where BCG vaccination is administered at birth, the optimal timing relative to potential *Mtb* exposure is unclear, as maternal immune factors (including transplacentally transferred antibodies and breast milk components) may modulate neonatal γδ T cell responses. Third, TCR repertoire sequencing in septic patients revealed that organism-specific expansion of fetal-derived Vγ9Vδ2 T cells can occur in response to both HMB-PP-producing (E. coli) and HMB-PP-deficient (*S. aureus*) bacteria (35) suggesting the existence of as-yet-unidentified activation mechanisms beyond phosphoantigen recognition—raising the possibility that the neonatal Vγ9Vδ2 T cell response is more complex than a simple phosphoantigen-driven model predicts. These considerations highlight the need for dedicated clinical studies in neonatal populations before the neonatal vaccination window strategy can be confidently recommended.

## Vγ9Vδ2 T cell-targeted immunotherapeutic strategies

The characteristics of Vγ9Vδ2 T cells—including MHC-unrestricted cytotoxicity, high potential for cytokine release, tissue tropism, and early activation during infection and malignancy—render them promising candidate cells for immunotherapy. Current immunotherapeutic strategies targeting Vγ9Vδ2 T cells can be broadly categorized into three approaches (1): pharmacological activation of endogenous Vγ9Vδ2 T cells using synthetic phosphoantigens or aminobisphosphonates (2); targeted drug development against BTN family molecules to enhance or modulate Vγ9Vδ2 T cell activation; and (105) adoptive transfer of ex vivo-expanded Vγ9Vδ2 T cells into patients (138). Each strategy presents distinct advantages and challenges, as discussed below (**Table 3**).

**Table 3:** Immunotherapy strategies targeting Vγ9Vδ2 T cells.

#	Strategy	Key antigen/component	Vγ9Vδ2 T cell response	Model system	Key findings	Reference
1	ZOL/IL-2 adjunctive immunotherapy (NHP)	Zoledronate (endogenous IPP accumulation) + rIL-2 + second-line anti-TB drugs	Vγ2Vδ2 T effector expansion with sustained IFN-γ/TNF-α production; enhanced CD4^+^/CD8^+^ Th1 populations trafficking to airways	NHP (multidrug-resistant tuberculosis (MDR-TB)-infected macaques)	Adjunctive ZOL/IL-2 reduced MDR-TB bacterial burden and pathology vs anti-TB drugs alone; sustained Vγ2Vδ2 effector function until week 21	(139)
2	Allogeneic Vγ9Vδ2 T cell therapy (clinical)	Ex vivo-expanded allogeneic Vγ9Vδ2 T cells (1×10^8^ cells/infusion × 12 courses, q2w)	Engraftment and functional persistence; promoted immune reconstitution	Human (MDR-TB patients, n=8, open-label single-arm pilot)	Safe (96 infusions, zero adverse events); promoted pulmonary lesion repair on CT; 7/8 patients achieved sputum conversion; partially improved host immunity	(140)
3	ZOL/IL-2 *in vivo* expansion (clinical, ongoing)	Zoledronate + rIL-2 (*in vivo* Vγ2Vδ2 expansion) + anti-TB chemotherapy	*In vivo* Vγ2Vδ2 T cell expansion and activation	Human (Phase 4, NCT05493267, n=30, randomized, open-label, enrolling)	Ongoing randomized controlled trial at Shanghai Pulmonary Hospital; evaluating ZOL/IL-2 adjunctive immunotherapy for MDR-TB	NCT05493267
4	Autologous Vγ9Vδ2 T cell adoptive transfer (NHP)	Ex vivo phosphoantigen/IL-2-activated autologous Vγ9Vδ2 T cells	Rapid migration to airways and lungs; EM/CM phenotype; IFN-γ/TNF-α production; anti-*Mtb* effector function	NHP (rhesus macaques)	Adoptively transferred Vγ9Vδ2 T cells rapidly trafficked to pulmonary compartments; expressed central/effector memory phenotype; produced anti-*Mtb* cytokines; limited *Mtb* dissemination	(40)
5	HMB-PP + Vitamin C + rIL-2 expansion	HMB-PP (50 nM) + Vitamin C (70 μM) + rIL-2	Enhanced Vγ9Vδ2 T cell expansion (EM 75-90%, CM 10-20% by day 14); purity ≥90%	*In vitro* (human PBMCs + THP-1/H37Rv macrophage infection model)	Effectively inhibited intracellular H37Rv growth in cell-contact-dependent manner; upregulated TNF-α/IFN-γ, downregulated IL-10/IL-17A	(141)

### Activation of endogenous Vγ9Vδ2 T cells

In recent years, researchers have been exploring the activation of Vγ9Vδ2 T cells by modulating endogenous phosphoantigen levels through synthetic phosphoantigens or pharmacological agents, thereby enhancing their roles in anti-infectious and antitumor immune responses. For instance, amino-bisphosphonate drugs such as risedronate and zoledronate can increase intracellular IPP concentrations (142), thereby activating Vγ9Vδ2 T cells; these agents are commonly used clinically for the treatment of certain cancers or osteoporosis (143–148). Alternatively, synthetic phosphoantigen analogues, such as bromohydrin pyrophosphate (BrHPP), act as direct agonists of the Vγ9Vδ2 TCR, mimicking natural phosphoantigens to directly activate Vγ9Vδ2 T cells rather than by enhancing endogenous phosphoantigen production (149, 150). This mechanism is distinct from that of aminobisphosphonates, which increase intracellular IPP accumulation by inhibiting the mevalonate pathway.

Bennouna et al. employed the synthetic phosphoantigen BrHPP in combination with low-dose IL-2, demonstrating favorable safety and tolerability profiles while inducing effective γδ T lymphocyte expansion in patients (151). Certain pAgs originate from extracellular sources and require a mechanism for translocation across the plasma membrane into the cell; studies have identified that ATP-binding cassette transporter A1 (ABCA1) can cooperate with apolipoprotein A-I and BTN3A1 to mediate extracellular release of pAgs from dendritic cells (152).

The *in vivo* efficacy of aminobisphosphonate-based Vγ9Vδ2 T cell activation has been evaluated in both preclinical and clinical settings. In an MDR-TB-infected NHP model, adjunctive administration of zoledronate and rIL-2 alongside second-line anti-TB drugs induced Vγ2Vδ2 T effector expansion with sustained IFN-γ and TNF-α production, enhanced CD4^+^ and CD8^+^ Th1 populations trafficking to airways, and reduced MDR-TB bacterial burden and pathology compared with anti-TB drugs alone, with Vγ2Vδ2 effector function persisting until at least week 21 post-treatment (139). This preclinical evidence has been translated into an ongoing Phase 4 randomized, open-label clinical trial (NCT05493267) evaluating ZOL/IL-2 adjunctive immunotherapy for MDR-TB patients (n=30) at Shanghai Pulmonary Hospital.

Liu et al. (141) reported an optimized ex vivo expansion protocol combining HMB-PP (50 nM) with Vitamin C (70 μM) and rIL-2, yielding enhanced Vγ9Vδ2 T cell expansion with an effector memory phenotype (75-90% EM, 10-20% CM by day 14) and ≥90% purity. These expanded Vγ9Vδ2 T cells effectively inhibited intracellular H37Rv growth in a THP-1 macrophage infection model in a cell-contact-dependent manner, upregulating TNF-α/IFN-γ while downregulating IL-10/IL-17A. This protocol provides a refined strategy for generating therapeutically potent Vγ9Vδ2 T cells for both research and potential adoptive transfer applications.

Due to the instability and polar nature of the pyrophosphate moiety in pAgs, researchers have recently synthesized phosphoantigen prodrugs (ProPAgens) (136), improving the drug-like properties of pAgs to facilitate Vγ9Vδ2 T cell activation. Concurrently, the efficacy of natural phosphoantigens is constrained by their low cellular permeability and metabolic instability; therefore, the development of more potent synthetic phosphoantigen prodrug forms holds significant importance. Harmon et al. employed organic synthetic chemistry methods to protect the cis-allylic alcohol of phosphoantigens as acetate esters, which exhibited higher activity than natural phosphoantigens in terms of γδ T cell proliferation and cytokine production (137).

Despite these promising preclinical results, the ZOL/IL-2 clinical translation faces substantial limitations that must be acknowledged. The NHP study (139), while demonstrating proof-of-concept, employed a relatively small number of animals (n=6 per group) without blinding, and the MDR-TB infection model in macaques—while informative—may not fully recapitulate the complexity of human MDR-TB disease, particularly with respect to the heterogeneous spectrum of drug resistance patterns and pulmonary pathology seen in patients. The ongoing Phase 4 trial (NCT05493267) represents an important step toward clinical validation; however, several design considerations warrant attention: (i) the sample size (n=30) may be underpowered to detect clinically meaningful differences in hard endpoints such as treatment failure or relapse; (ii) the open-label design introduces potential bias in radiological outcome assessment; and (iii) the long-term safety of repeated ZOL administration combined with IL-2—including potential autoimmune complications, renal toxicity from ZOL, and IL-2-associated vascular leak syndrome—remains uncharacterized in the TB patient population. These concerns underscore the need for larger, blinded, placebo-controlled trials with adequate follow-up duration before definitive efficacy conclusions can be drawn.

### Adoptive Vγ9Vδ2 T cell transfer

Adoptive transfer of ex vivo-activated Vγ9Vδ2 T cells represents a complementary immunotherapeutic approach. In an NHP model, Qaqish et al. demonstrated that autologous Vγ9Vδ2 T cells activated ex vivo by phosphoantigen and IL-2 rapidly trafficked to airways and lungs following adoptive transfer, expressed central/effector memory phenotype, produced IFN-γ and TNF-α, and exhibited anti-*Mtb* effector function with limited *Mtb* dissemination (40). An immunotherapeutic approach based on allogeneic Vγ9Vδ2 T cells was evaluated for safety and efficacy in patients with multidrug-resistant tuberculosis (MDR-TB); results demonstrated that clinical administration of Vγ9Vδ2 T cells was safe (96 infusions, zero adverse events), with clinical outcomes including reduction of *in vivo Mtb* burden, promotion of pulmonary lesion repair on CT imaging, and partial improvement of host immunity (153), thereby providing preliminary evidence for Vγ9Vδ2 T cell-based immunotherapy in MDR-TB. However, as this study was an open-label, single-arm pilot trial with a small sample size (n=8), these findings require validation in larger randomized controlled trials before definitive efficacy conclusions can be drawn. Patient responses to these treatments vary considerably, with some exhibiting favorable outcomes while others achieve only modest improvement. This heterogeneity may be attributable to the limited understanding of γδ TCR diversity and the mode of receptor–ligand interactions, or potentially to autologous killing of Vγ9Vδ2 T cells resulting from self-activation by exogenous phosphoantigens (62).

The adoptive transfer approach for MDR-TB faces several important limitations that extend beyond the small sample size. First, the allogeneic nature of the transferred cells raises the question of persistence: allogeneic Vγ9Vδ2 T cells may be rejected by the host immune system, limiting the duration of therapeutic effect. While the pilot study reported no adverse events (153), the absence of long-term engraftment data means that the sustainability of the therapeutic effect remains unproven. Second, the manufacturing process—ex vivo expansion to ≥90% purity—introduces batch-to-batch variability that may compromise reproducibility across clinical sites. The field lacks standardized Good Manufacturing Practice (GMP)-compliant protocols for Vγ9Vδ2 T cell expansion suitable for multi-center clinical trials. Third, the functional quality of ex vivo-expanded cells may differ from that of naturally activated cells: expansion under artificial conditions (high-dose IL-2, phosphoantigen stimulation) may drive cells toward terminal effector differentiation at the expense of memory potential, potentially limiting long-term protective efficacy. Finally, the cost-effectiveness of adoptive Vγ9Vδ2 T cell therapy compared with newer anti-TB drug regimens has not been formally evaluated—an important consideration for deployment in low- and middle-income countries where TB burden is highest.

### Targeted drug development

BTN3A1 and BTN2A1 may provide potential intervention targets for the development of novel tuberculosis immunotherapeutic strategies. Treatment of target cells with the agonistic BTN3A1-specific monoclonal antibody (mAb) 20.1 mimics pAg-induced Vγ9Vδ2 T cell activation (154); conversely, exposure of *Mtb*-infected target cells to the antagonistic mAb 103.2 abrogates Vγ9Vδ2 T cell activation (155). Furthermore, the intracellular B30.2 domain of BTN3A1 directly binds phosphoantigens through a positively charged surface pocket to mediate activation of human Vγ9Vδ2 T cells (156). Recently, De Gassart et al. (157) developed a humanized anti-BTN3A monoclonal antibody (ICT01) that activates Vγ9Vδ2 T cells to promote augmented immune responses through production of proinflammatory cytokines (e.g., IFN-γ and TNF-α), with favorable tolerability in cynomolgus monkeys; the ICT01 antibody represents a promising clinical strategy. Payne et al. (32) proposed that anti-CD277 antibodies can relieve BTN3A1-mediated inhibition of αβ T cells and synergistically enhance γδ T cell activity to improve the potential of tumor immunotherapy. Du et al. (158) investigated the role of BTNL2 in antitumor immunity through animal models and clinical samples, demonstrating that BTNL2 interacts with γδ T cells to promote IL-17A production in the tumor microenvironment, thereby increasing tumor resistance to immunity and exhibiting a negative correlation with survival in clinical patients.

While these BTN-targeted strategies have shown promise in oncology, their extrapolation to tuberculosis requires careful consideration. The tumor microenvironment and TB granuloma present fundamentally distinct immunological contexts: tumors are characterized by immunosuppressive checkpoints and metabolic competition, whereas TB granulomas feature necrotic cores, hypoxia, and potential Mtb-driven impairment of BTN3A1 expression, which has been associated with but not yet mechanistically attributed to epigenetic silencing (101). Notably, the antagonistic antibody 103.2, which abrogates Vγ9Vδ2 T cell activation in *Mtb*-infected cells (155), underscores that BTN3A1 signaling is actively suppressed during TB infection—raising the question of whether agonistic antibodies alone can overcome this pathogen-driven inhibition. By enhancing BTN3A1/BTN2A1-mediated Vγ9Vδ2 T cell activation signals, it may be possible to overcome *Mtb*-induced immunosuppressive microenvironments and restore functionality in exhausted T cells; however, preclinical validation of BTN-targeted approaches in TB infection models is essential before clinical translation. Combined use of BTN agonistic antibodies with phosphoantigen prodrugs or with agents that reverse *Mtb*-induced BTN3A1 epigenetic silencing may generate synergistic effects, offering potential options for the treatment of drug-resistant tuberculosis.

The extrapolation of BTN-targeted strategies from oncology to TB faces several fundamental obstacles. First, ICT01 was developed and tested in solid tumor contexts (157); its pharmacokinetics, biodistribution, and safety profile in the context of chronic infectious disease with active granulomatous inflammation remain entirely uncharacterized. Second, the hypoxic and caseous environment of TB granulomas may impair antibody penetration and access to BTN3A1-expressing cells within the lesion core—a challenge not encountered in the relatively well-vascularized tumor microenvironment. Third, while agonistic BTN3A1 antibodies can activate Vγ9Vδ2 T cells in the tumor context, the risk of exacerbating immunopathology in TB must be carefully considered: uncontrolled Vγ9Vδ2 T cell activation in the lung could drive excessive inflammation, tissue damage, and potentially facilitate cavitation—a major driver of TB transmission. This tension between immunostimulation and immunopathology represents a unique challenge for BTN-targeted TB therapy that has no parallel in oncology. Fourth, the observation that Vγ9Vδ2 T cell anergy in active TB is associated with reduced responsiveness that may involve BTN3A1 downregulation (101) suggests that simply providing exogenous agonistic signals may be insufficient if the endogenous antigen presentation machinery is fundamentally impaired. Whether this impairment is mediated by epigenetic mechanisms remains to be formally demonstrated. Combinatorial approaches integrating BTN agonistic antibodies with epigenetic modulators (e.g., histone deacetylase inhibitors that may reverse BTN3A1 silencing) represent a rational but experimentally unexplored strategy.

## Conclusions and perspectives

Vγ9Vδ2 T cell biology in TB encompasses distinct developmental programs, multifaceted activation mechanisms, and diverse effector functions that position these cells as promising targets for immunotherapy and vaccine development. However, several fundamental challenges must be addressed to advance translational progress. While the potential of Vγ9Vδ2 T cells in tuberculosis control is evident from preclinical studies, several fundamental challenges demand systematic attention.

Several key gaps must be addressed:

1. The absence of longitudinal studies tracking Vγ9Vδ2 T cell dynamics from *Mtb* infection through disease progression and treatment. Notably, the majority of evidence supporting current models comes from cross-sectional studies comparing distinct patient groups (healthy controls, latent TB, active TB); longitudinal analyses tracking individual Vγ9Vδ2 T cell populations from infection through disease progression are conspicuously absent. Such studies are essential to establish causal relationships between Vγ9Vδ2 T cell functional changes and TB outcomes, and to define correlates of protection suitable for vaccine development.
2. Unresolved heterogeneity of Vγ9Vδ2 T cell subsets across infection stages and anatomical compartments. The paradoxical Vδ2→Vδ1 shift during chronic infection, the distinct transcriptional programs of Vγ9Vδ2 T cells in blood versus lung tissue, and the functional heterogeneity revealed by single-cell transcriptomics all point to a complexity that has been inadequately addressed by bulk analysis approaches. Current studies typically characterize Vγ9Vδ2 T cells as a homogeneous population, yet emerging single-cell data reveal substantial functional diversity (111) that may have profound implications for therapeutic targeting.
3. The molecular and epigenetic mechanisms of *Mtb*-driven Vγ9Vδ2 T cell functional exhaustion remain poorly characterized. While phenotypic descriptions of exhaustion (PD-1 upregulation, cytokine impairment) are well-documented (101, 126), the underlying epigenetic reprogramming—including DNA methylation changes, histone modifications, and chromatin accessibility alterations at key effector gene loci—has not been systematically investigated in Vγ9Vδ2 T cells. Understanding these mechanisms is prerequisite to developing strategies that reverse exhaustion and restore functionality.
4. The lack of standardized methodologies for assessing Vγ9Vδ2 T cell responses across studies. Inconsistent gating strategies, variable ex vivo expansion protocols, diverse readout assays, and the absence of internationally recognized reference standards hinder cross-study comparisons and meta-analyses. The field urgently needs consensus guidelines for the characterization of Vγ9Vδ2 T cell responses in TB research, analogous to those developed for HIV-specific T cell responses.
5. The limited preclinical validation of BTN-targeted strategies in TB-specific infection models. While BTN-targeted antibodies have reached clinical trials in oncology (157), no preclinical study has evaluated their efficacy in the context of *Mtb* infection. The fundamental differences between the tumor microenvironment and the TB granuloma necessitate dedicated validation before clinical translation can be considered.
6. The distinction between ‘trained immunity’ and ‘immunological memory’ in γδ T cells remains incompletely defined, with profound implications for vaccine design. The distinction between antigen-specific memory and non-specific innate reprogramming has profound implications for vaccine design strategies, yet current evidence is insufficient to definitively resolve this debate. Rigorous experimental approaches—including epigenetic profiling, metabolic flux analysis, and long-term *in vivo* tracking of clonally barcoded Vγ9Vδ2 T cells—are needed to distinguish between these models.

Emerging technologies offer transformative opportunities to address these gaps. Recent advances in single-cell multi-omics (scRNA-seq, scATAC-seq, CITE-seq) and spatial transcriptomics have begun to reveal the extraordinary heterogeneity of γδ T cell populations at single-cell resolution and within their tissue context (41, 111). The convergence of CRISPR-based screening with γδ T cell biology represents a promising frontier for identifying key regulatory genes governing Vγ9Vδ2 T cell effector function, exhaustion, and memory formation. Epigenetic profiling through ATAC-seq and bisulfite sequencing in sorted Vγ9Vδ2 T cell subsets from defined TB disease states could reveal the chromatin-level changes underlying functional impairment and inform the development of epigenetic intervention strategies. Additionally, the development of advanced *in vitro* models—including three-dimensional granuloma organoids incorporating Vγ9Vδ2 T cells and humanized mouse models with functional Vγ9Vδ2 T cell compartments—would enable mechanistic studies that bridge the gap between *in vitro* findings and *in vivo* relevance.

Based on this comprehensive analysis, we propose a research roadmap for advancing Vγ9Vδ2 T cell-based TB interventions:

1. Short-term (1–3 years): Establish standardized protocols for Vγ9Vδ2 T cell characterization across laboratories; conduct pilot spatial transcriptomic studies of γδ T cells within human TB granulomas; initiate longitudinal cohort studies with serial Vγ9Vδ2 T cell profiling from LTBI through active TB; validate BTN agonistic antibody efficacy in established TB infection models in humanized mice.
2. Medium-term (3–5 years): Complete Phase 4 RCT of ZOL/IL-2 adjunctive therapy with adequate power and blinded outcome assessment; develop GMP-compliant manufacturing protocols for clinical-grade Vγ9Vδ2 T cell expansion; perform comprehensive epigenetic profiling of Vγ9Vδ2 T cells across the TB disease spectrum; evaluate combinatorial approaches (BTN agonistic antibodies + phosphoantigen prodrugs + epigenetic modulators) in NHP TB models.
3. Long-term (5–10 years): Design and initiate Vγ9Vδ2 T cell-enhanced vaccine trials based on validated correlates of protection; establish CRISPR-based functional screens to identify novel therapeutic targets in Vγ9Vδ2 T cells; develop personalized immunotherapy protocols for MDR-TB patients incorporating Vγ9Vδ2 T cell expansion, phenotypic quality control, and functional validation.

Through interdisciplinary collaboration integrating systems immunology, epigenetics, spatial biology, and clinical trial methodology, it is anticipated that research advances in Vγ9Vδ2 T cells can be translated into effective tools for tuberculosis prevention and control, contributing to the global endeavor to end the tuberculosis epidemic.
